# Discovery of 7‑(Pyridin-3-yl)thieno[3,2‑*b*]pyridine-5-carboxamides as Negative Allosteric Modulators of Metabotropic Glutamate Receptor Subtype 5

**DOI:** 10.1021/acschemneuro.5c00896

**Published:** 2026-01-26

**Authors:** Scott H. Henderson, Anna E. Ringuette, David L. Whomble, Rory A. Capstick, Alexa E. Richardson, Mallory A. Maurer, Natasha B. Billard, Xia Lei, Joshua C. Wilkinson, Sri H. Kethanapalli, Hyekyung P. Cho, Alice L. Rodriguez, Colleen M. Niswender, Weimin Peng, Jerri M. Rook, Sichen Chang, Anna L. Blobaum, Olivier Boutaud, Andrew S. Felts, P. Jeffrey Conn, Craig W. Lindsley, Kayla J. Temple

**Affiliations:** † Warren Center for Neuroscience Drug Discovery, 5718 Vanderbilt University, Nashville, Tennessee 37232, United States; ‡ Department of Pharmacology, 12327 Vanderbilt University School of Medicine, Nashville, Tennessee 37232, United States; § Department of Chemistry, Vanderbilt University, Nashville, Tennessee 37232, United States; ∥ Department of Biochemistry, Vanderbilt University, Nashville, Tennessee 37232, United States; ⊥ Vanderbilt Kennedy Center, 12328 Vanderbilt University Medical Center, Nashville, Tennessee 37232, United States; # Vanderbilt Brain Institute, Vanderbilt University School of Medicine, Nashville, Tennessee 37232, United States; ∇ Vanderbilt Institute of Chemical Biology, Vanderbilt University School of Medicine, Nashville, Tennessee 37232, United States; ○ Vanderbilt Institute for Therapeutic Advances, 5718 Vanderbilt University, Nashville, Tennessee 37232, United States

**Keywords:** metabotropic glutamate receptor subtype 5, mGlu_5_, negative allosteric modulator, structure−activity relationship, leveodopa-induced dyskinesia, Alzheimer’s, pain, VU6035386

## Abstract

Herein, we report the structure–activity relationship (SAR) to develop novel mGlu_5_ negative allosteric modulator (NAM) scaffolds devoid of the aryl/heterobiaryl acetylene moiety found in many historic mGlu_5_ NAMs, which has been linked to metabolic liabilities and hepatotoxicity. This endeavor utilized a scaffold-hopping strategy from the predecessor compound **VU6031545**, in which we replace an ether-linked tetrahydrofuran with various carbon-linked heteroaryl motifs to generate highly potent and selective mGlu_5_ NAMs. One such compound, **VU6035386**, displayed low nanomolar potency against human mGlu_5_ and was highly brain penetrant. Moreover, **VU6035386** showed a vast improvement in predicted human hepatic clearance versus predecessor compound **VU6031545**.

## Graphical abstract

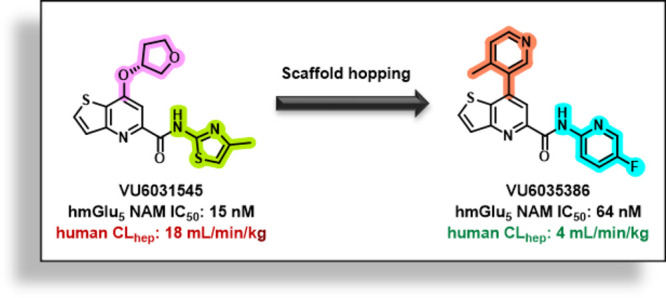

## Introduction

The metabotropic glutamate receptors (mGlu receptors) are a family of eight G-protein-coupled receptors (GPCRs). Once activated by l-glutamic acid, the major excitatory neurotransmitter of the mammalian central nervous system (CNS), the mGlu receptors modulate the strength of synaptic transmission. The mGlu receptors are classified into three groups based not only on structure and sequence homology, but also on pharmacology and downstream signaling partners/pathways. Widely expressed throughout the CNS, the metabotropic glutamate receptor 5 (mGlu_5_) belongs to group I mGlu receptors (alongside mGlu_1_) which are predominantly found postsynaptically and activate phospholipase C (PLC) via G_q_ coupling.^1,2^ Due to the highly conserved nature of the orthosteric glutamate site, ligands that modulate receptor function via orthosteric binding exhibit poor subtype selectivity among the mGlu receptors resulting in increased risk of off-target adverse events (AEs). Our laboratory utilizes allosteric modulation, which has proven to be a successful approach to selectively target individual mGlu receptor subtypes. Since the discovery of the selective mGlu_5_ antagonist 2-methyl-6-(phenylethynyl)-pyridine (MPEP, **1**; Figure 1) over two decades ago, mGlu_5_ negative allosteric modulators (NAMs) have become some of the most advanced and extensively investigated within the field of metabotropic glutamate receptor allostery.^3–8^

**Figure 1:**
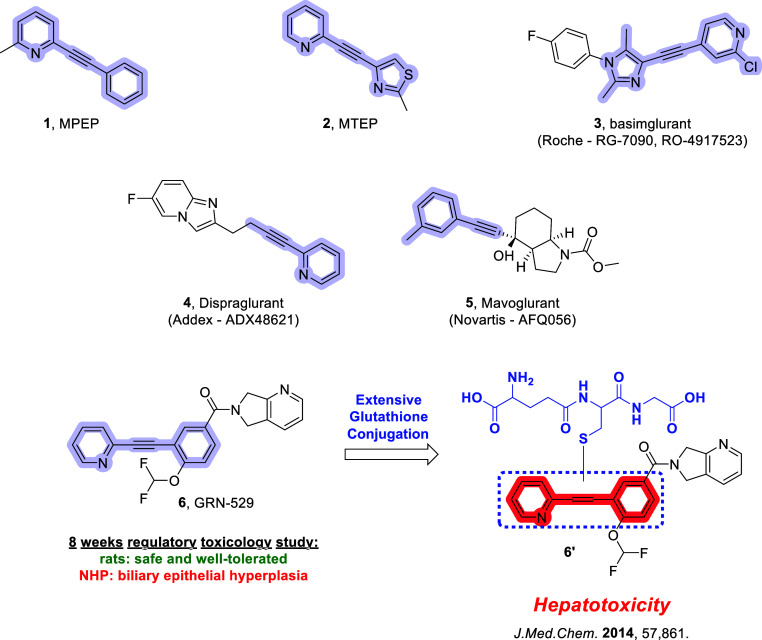
Prototypical, acetylene-based mGlu_5_ NAM chemotypes. NAMs **1** and **2** were crucial early tool compounds and led to the development of NAMs **3–6**, which entered human clinical testing. Extensive glutathione conjugation to the acetylene moiety in nonhuman primate (NHP) hepatic tissue, bile, and plasma samples strongly suggested a structural link to the observed hepatotoxicity.

Preclinical and clinical efficacy has substantiated an amassment of potential therapeutic areas for small molecule mGlu_5_ NAMs. Potential therapeutic applications include anxiety,^9,10^ Alzheimer’s disease (AD),^11^ fragile X syndrome (FXS),^12–14^ autism spectrum disorder (ASD),^15,16^ levodopa-induced dyskinesia (LID) associated with Parkinson’s disease (PD) patients,^17–19^ gastroesophageal reflux disease (GERD),^20^ addiction disorder,^21–23^ major depressive disorder (MDD),^24–26^ obsessive-compulsive disorder (OCD),^27^ migraine and pain.^28–31^ Despite promising preclinical data and progression into phase II clinical trials, many mGlu_5_ NAMs have largely failed due to safety concerns and/or lack of efficacy (possibly due to dose-limited toxicity).^32–34^ One possible explanation for these clinical failures is the key pharmacophore of early mGlu_5_ antagonists (e.g., **1** and **2,** Figure 1), a disubstituted heterobiaryl acetylene moiety. Acetylene-containing compounds have long been associated with metabolic liabilities and consequent hepatotoxicity. Alkynes, particularly those conjugated with an α-heteroatom, are potentially reactive functional groups.^35,36^ This potential toxicophore has been carried throughout several subsequent medicinal chemistry campaigns (e.g., **1–6,** Figure 1) and, as a result, many acetylene-based mGlu_5_ NAMs have been linked to hepatotoxicity and glutathione conjugation as reported in both preclinical and clinical studies.^37^

Employing an acetylene bioisostere to circumvent the toxicophore led to the discovery of AZD9272 (**8**) which was selected for clinical development (Figure 2). Fenobam (**7**), an mGlu_5_ NAM completely devoid of the aryl/heterobiaryl acetylene moiety, was also progressed into clinical trials (Figure 2). Unfortunately, both **7** and **8** were reported to induce psychosis-like AEs and their development was halted. Further investigation indicates that **7** and **8** bind to non-mGlu_5_-related sites, such as monoamine oxidase-B (MAO-B), which most likely accounts for the psychotic symptoms observed in early clinical trials.^38^ To date, no mGlu_5_ NAM has progressed to the market. This is partially attributed to dose-limiting AEs (i.e., hallucinations or psychotomimetic effects) observed in some clinical trials.^39^ TMP-301 (**9**) is currently undergoing clinical trials for substance abuse disorders; however, due to the high structural conservation with AZD9272 (**8**), TMP-301 may pose similar AEs.^40^ To exploit the broad therapeutic utility of a selective mGlu_5_ NAM, focus in the field has shifted to identifying novel, nonacetylene containing mGlu_5_ NAMs in an effort to overcome pharmacophore-mediated adverse liabilities.

**Figure 2:**
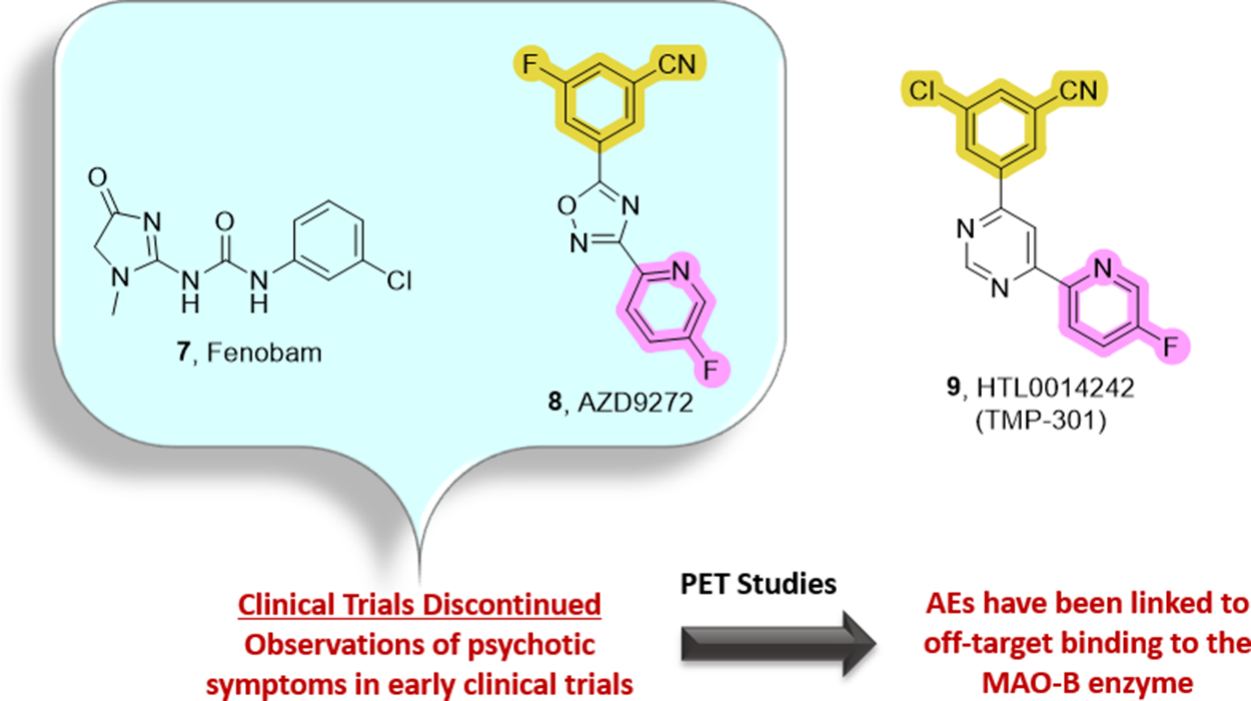
Nonacetylene-based mGlu_5_ NAMs advanced into clinical trials. Fenobam (**7**) and AZD9297 (**8**) development was discontinued due to adverse effects linked to off-target binding at the MAO-B enzyme.

The development of small molecule mGlu5 NAMs has been a major area of research in our laboratory. Our efforts culminated in the identification of clinical candidate **10** (auglurant, **VU0424238**) (Figure 3).^41^ During a 28-day toxicological assessment in cynomolgus monkeys, species-specific toxicities were observed. Although no such observations were previously noted in rats, further development of **10** was halted. A more in-depth evaluation revealed accumulation of an aldehyde oxidase (AO) metabolite, which was only observed at 14 days and resulted in pronounced anemia (nonmechanism-based).^42^ Metabolic assessment revealed oxidation of the pyrimidine ring to a 6-oxopyrimidine metabolite which underwent further metabolism to subsequently form a 2,6-oxopyrimidine metabolite.

**Figure 3:**
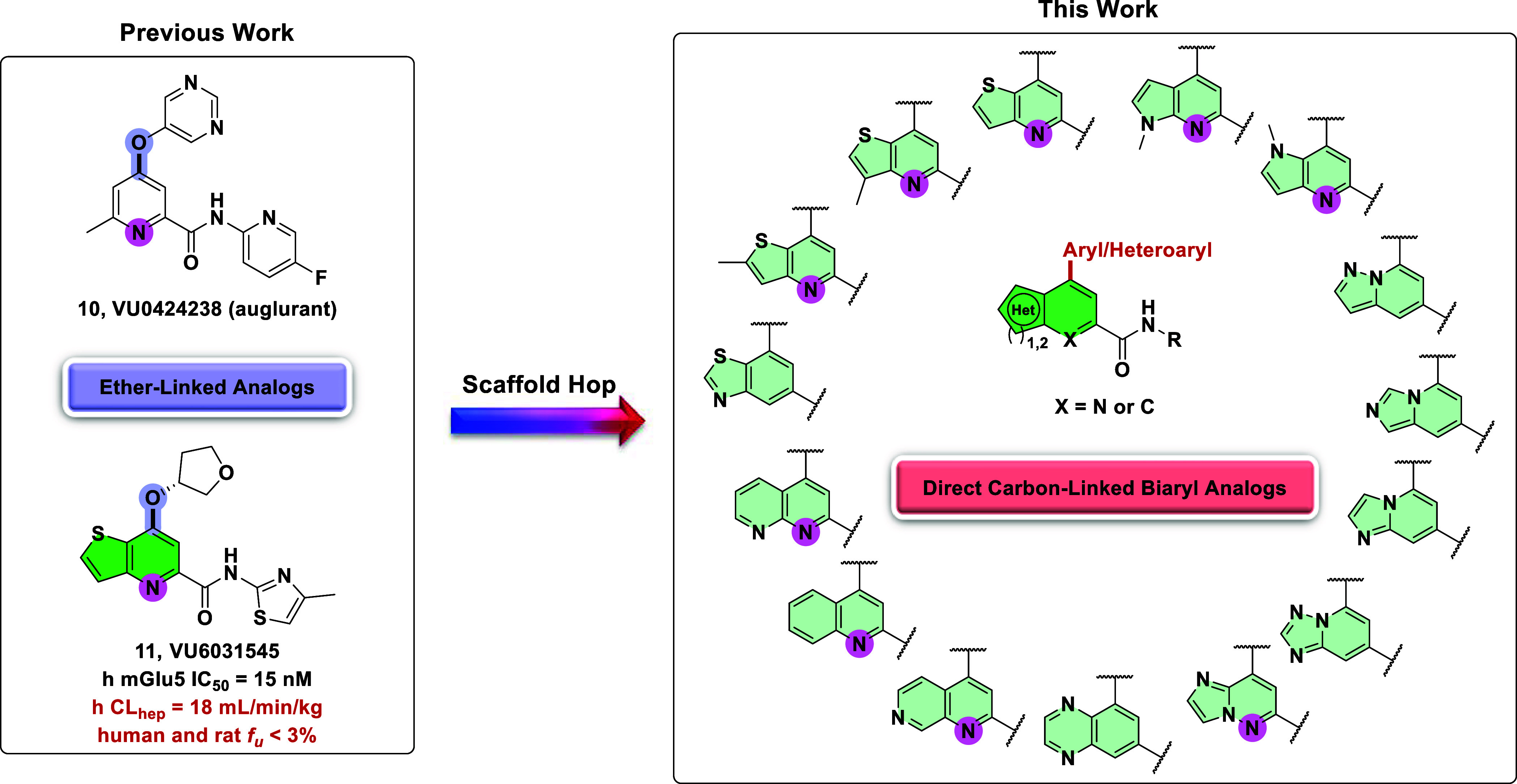
Previously published compounds that emerged from optimization of HTS hits: clinical candidate **VU0424238** (auglurant, **10**) and backup scaffold **11**. A scaffold hopping exercise led to the discovery of direct carbon-linked biaryl mGlu_5_ NAMs.

While the formation of the 6-oxopyrimide metabolite was determined to be mediated by AO in rat, monkey, and human, species differences between rats and monkeys were noted in the subsequent metabolism step. In monkeys, the formation of the 2,6-oxopyrimidine metabolite was determined to be AO-mediated; conversely, the same metabolic transformation was mediated by xanthine oxidase (XO) metabolism in rats.^42,43^ Therefore, it is possible that species differences in the involvement of AO/XO metabolism may play a role in the observed monkey-specific toxicity. As a result, attention was shifted to the development of backup analogs, such as **VU6031545** (**11**), to identify a compound devoid of AO metabolism by elimination of the pyrimidine liability.^44^ While compound **11** was highly potent (human mGlu_5_ IC_50_ = 15 nM), it suffered from high predicted human hepatic clearance. Thus, further optimization was required. This letter describes a scaffold-hopping exercise that subsequently led to the discovery of novel mGlu_5_ NAMs containing direct carbon-linked biaryl/heteroaryl motifs (Figure 3).

## Results and Discussion

To synthesize analogs **25**, we first focused on generating noncommercial thieno­[3,2-*b]*pyridine intermediates **17–19** (Scheme 1). To begin, commercially available chlorides **12** underwent standard oxidation conditions to afford pyridine-*N*-oxides **13–15**. Trimethylsilyl cyanide was then utilized, with dimethylcarbamoyl chloride as the activating electrophile, to convert intermediates **13–15** into the 2-cyanopyridines **17–19** in high yields. Similarly, commercially available 7-chloro-1*H*-pyrrolo­[3,2-*b*]­pyridine could undergo the same sequence of transformations to afford intermediate **20** which was further reacted with sodium hydride and methyl iodide to give intermediate **21**. Intermediate **23** was commercially available while intermediate **22** could be synthesized according to literature protocols.^45^

**Scheme 1:**
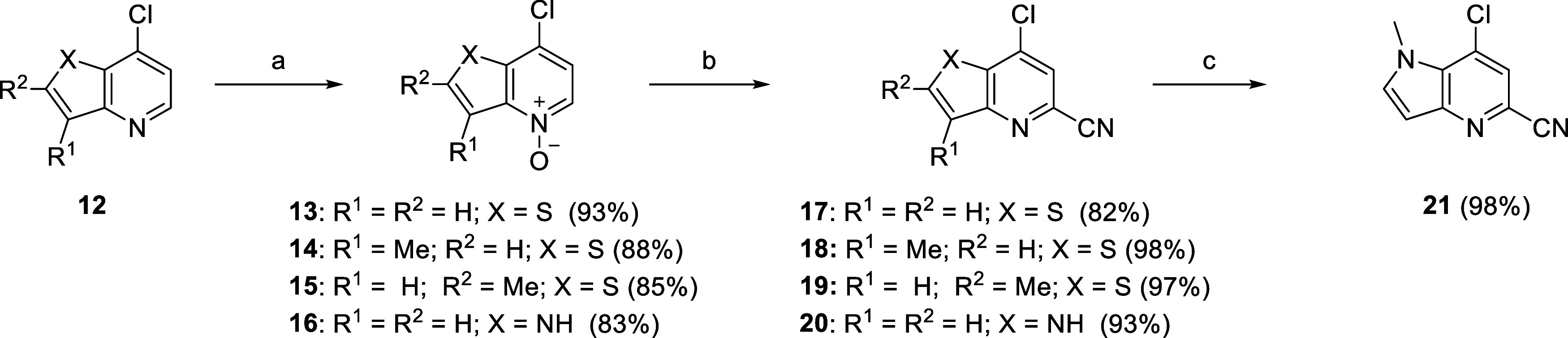
Synthesis of mGlu_5_ NAM Intermediates **17–21** ^a^

a Reagents and conditions: (a) *m*-CPBA, DCM; (b) TMSCN, DMCC, DCM; and (c) NaH, MeI, DMF, 0 °C.

Undergoing a direct scaffold-hop from analog **11**, we prioritized the *N*-(4-methylthiazol-2-yl)­thieno­[3,2-*b*]­pyridine-5-carboxamide core. Thus, nitrile **17** underwent basic hydrolysis to carboxylic acid **27** (Scheme 2, Route 3). Subsequent conversion to the acid chloride and reaction with 4-methylthiazol-2-amine afforded intermediate **28** which was further reacted using standard palladium cross-coupling conditions to afford the pinacol borane **29**. Intermediate **29** could then undergo Suzuki couplings with various aryl/heteroaryl halides to afford analogs **30,** which were tested at human mGlu_5_ (hmGlu_5_) to determine potency with results highlighted in Table 1. In general, both 3-pyridyl (**30f**: hmGlu_5_ IC_50_ = 20 nM) and 4-pyridyl (**30c**: hmGlu_5_ IC_50_ = 46 nM) groups were well tolerated and provided some of the most potent compounds (hmGlu_5_ IC_50_ < 50 nM). When substituting the 3-pyridyl group *para* to the bridging carbon, only a strong electron withdrawing group (EWG) was tolerated (**30j**: hmGlu_5_ IC_50_ = 195 nM). Conversely, substitution at the *para* position with either a weak EWG (**30q**: hmGlu_5_ IC_50_ > 10 μM) or an electron donating group (EDG) (**30r**: hmGlu_5_ IC_50_ > 10 μM) resulting in a substantial loss of activity. Alternatively, when substituting the 3-pyridyl group *meta* to the bridging carbon, both EWGs (**30b**: hmGlu_5_ IC_50_ = 15 nM; **30i**: hmGlu_5_ IC_50_ = 85 nM) and EDG (**30h**: hmGlu_5_ IC_50_ = 31 nM) were well tolerated. Substituting the 3-pyridyl group *para* to the nitrogen of the pyridine also provided potent analogs (**30a**, **30g**, **30p**, and **30u**). While a methyl substitution at this position was tolerated (**30a**: hmGlu_5_ IC_50_ = 296 nM), the most potent of these analogs contained small EWGs such as a fluorine (**30p**: hmGlu_5_ IC_50_ = 25 nM) or a nitrile (**30g**: hmGlu_5_ IC_50_ = 62 nM). Interestingly, a drastic loss in potency was observed with the 2-pyridyl group (**30l**: hmGlu_5_ IC_50_ > 10 μM); however, activity could be recovered with the incorporation of an additional nitrogen into the ring to give pyrazine **30n** (hmGlu_5_ IC_50_ = 178 nM). These data highlight the importance of nitrogen’s orientation to the bridging carbon.

**Scheme 2:**
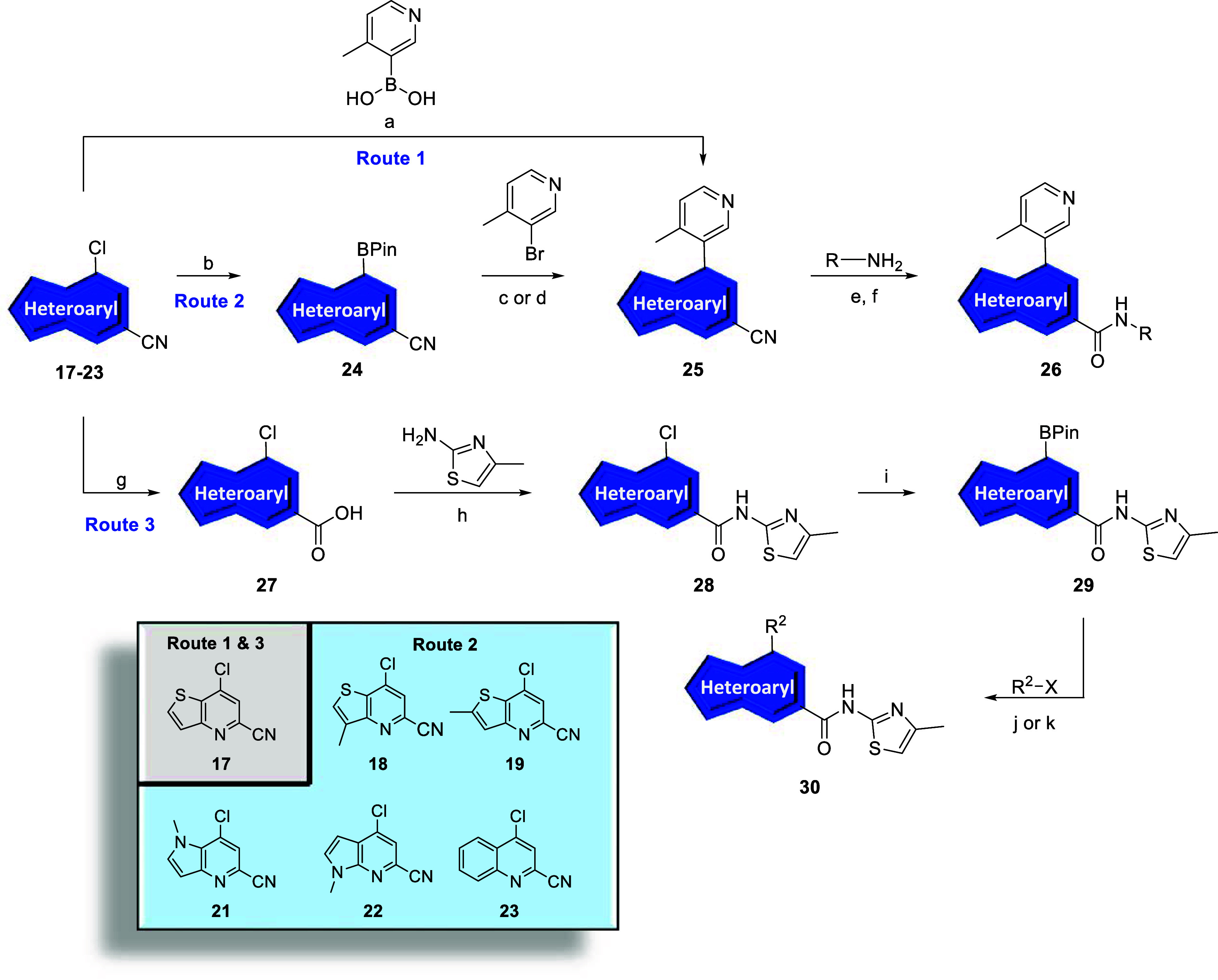
Synthesis of mGlu_5_ NAM Analogs **26** and **30** ^a^

a Reagents and conditions: (a) Pd­(dppf)­Cl_2_, Cs_2_CO_3_, 1,4-dioxanes, microwave irradiation 120 °C, 81%; (b) B_2_pin_2_, KOAc, PdCl_2_(PPh_3_)_2_, 1,4-dioxanes, 80 °C, 88–97%; (c) BrettPhos Pd G_3_, K_2_CO_3_, 1,4-dioxanes/water (9:1), 100 °C, 70–98%; (d) Pd­(dppf)­Cl_2_, Cs_2_CO_3_, MeCN/water (9:1), 80 °C, 68–98%; (e) NaOH_(aq)_, 1,4-dioxanes, 100 °C, 2–18 h, 68–98%; (f) amine, POCl_3_, pyridine, 0 °C to rt, 5–82%; (g) NaOH_(aq)_ (2*M*), 1,4-dioxanes, 100 °C, 2–18 h, 74%; (h) amine, POCl_3_, pyridine, 0 °C to rt, 74%; (i) B_2_pin_2_, KOAc, PdCl_2_(PPh_3_)_2_, 1,4-dioxanes, microwave irradiation 120 °C, 1 h, 61%; (j) Pd­(dppf)­Cl_2_, Cs_2_CO_3_, 1,4-dioxanes/water (3.3:1), microwave irradiation 120 °C, 0.25h, 4–23%; (k) Pd­(dppf)­Cl_2_, K_2_CO_3_, IPA/water/toluene (1:1:2), 95 °C, 10–97%.

**Table 1:**
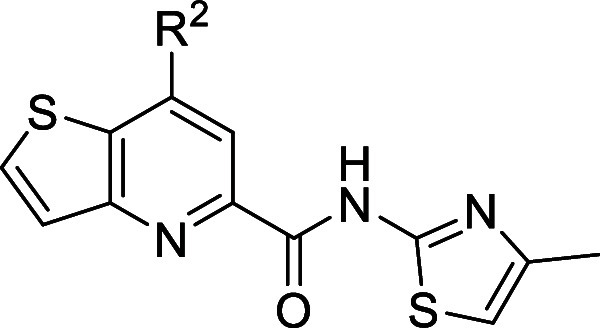

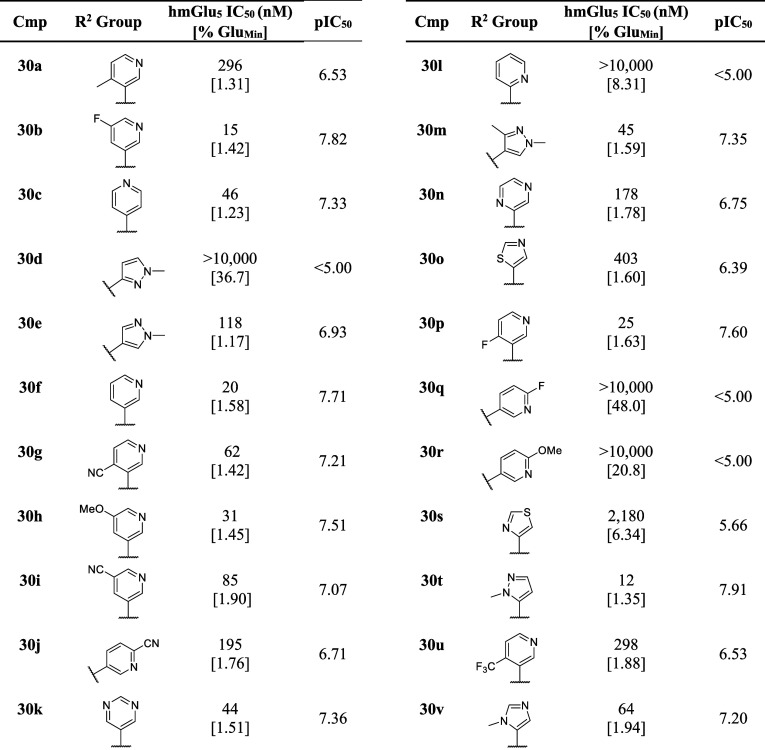
Structures and Activities for Thieno­[3,2-*b*]­pyridine Analogs **30** ^a^

a Calcium mobilization assays in human mGlu_5_-HEK293A cells were performed in the presence of an EC_80_ fixed concentration of glutamate, *n* = 2 independent experiments in triplicate. The % Glu_Min_ is the measure of efficacy of the NAM to reduce an EC_80_ concentration of glutamate.

Moreover, 5-membered heterocyclic groups were also permissible as pyridine replacements. Although 1-methyl-1*H*-pyrazole was weakly active when linked via the 3-position of the pyrazole (**30d**: hmGlu_5_ IC_50_ > 10 μM), the regioisomers regained potency (**30e**: hmGlu_5_ IC_50_ = 118 nM; **30t**: hmGlu_5_ IC_50_ = 12 nM), once again emphasizing the importance of the nonalkylated nitrogen’s orientation to the bridging carbon. By adding a methyl to the pyrazole ring of **30e**, we were able to increase potency by more than 2.5-fold (**30m**: hmGlu_5_ IC_50_ = 45 nM). Exchanging the 1-methyl-1*H*-pyrazole ring of **30e** and replacing with a 1-methyl-1*H*-imidazole group also resulted in a nearly 2-fold increase in potency (**30v**: hmGlu_5_ IC_50_ = 64 nM). While thiazoles were also evaluated, the resulting analogs only provided modest potencies (**30o**: hmGlu_5_ IC_50_ = 403 nM; **30s**: hmGlu_5_ IC_50_ = 2.2 μM).

Using an early discovered potent mGlu_5_ NAM (**30a**), we began to test various amide groups in parallel (**26**, Scheme 2). To carry out the synthesis, chloride **17** was subjected to standard Suzuki-coupling conditions with commercially available boronic acids/esters to give nitrile **25** (Scheme 2, Route 1). Alternatively, chloride **17** could undergo standard palladium cross-coupling conditions to afford pinacol borane **24** which could be converted into nitrile **25** via a Suzuki cross-coupling with various commercially available aryl/heteroaryl halides (Scheme 2, Route 2). Intermediate **25** then underwent basic hydrolysis to the carboxylic acid which proceeded smoothly in 68–98% yields. Finally, conversion to the acid chloride and reaction with various amines *in situ* afforded analogs **26**. Analogs **26a**–**26x** were screened against human mGlu_5_ to determine potency with results highlighted in Table 2.

**Table 2:**
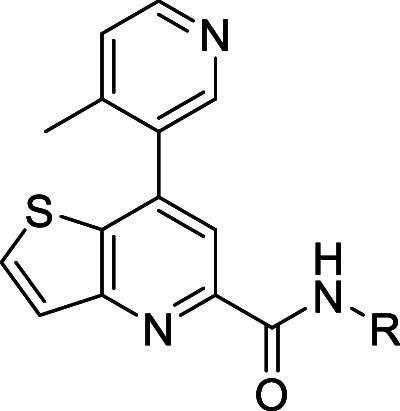

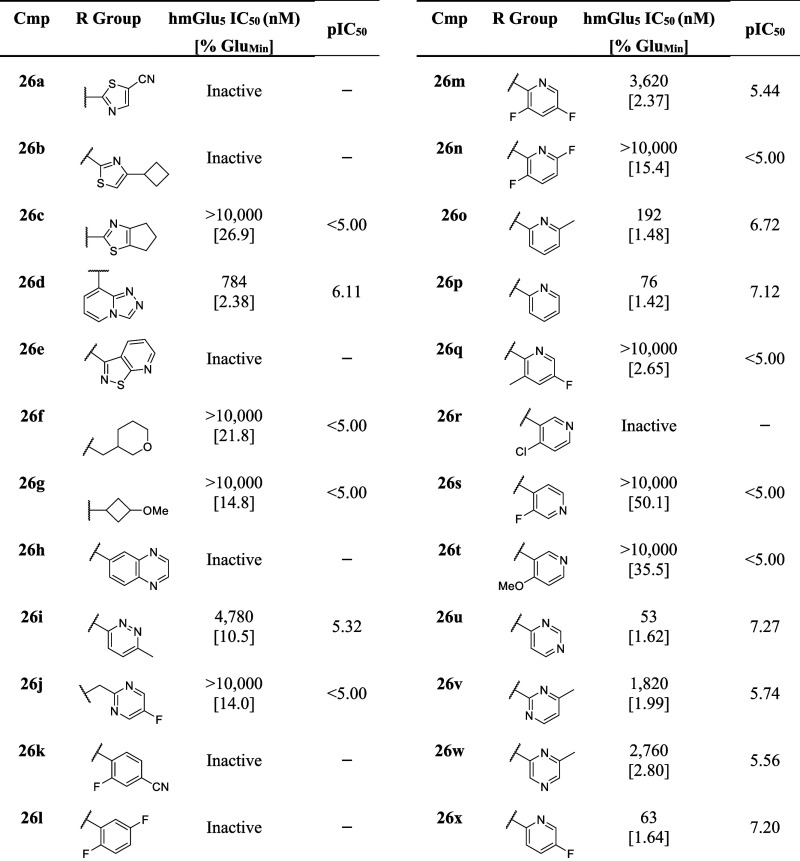
Structures and Activities for Thieno­[3,2-*b*]­pyridine Analogs **26a**–**26x** ^a^

a Calcium mobilization assays in human mGlu_5_-HEK293A cells were performed in the presence of an EC_80_ fixed concentration of glutamate, *n* = 2 independent experiments in triplicate. The % Glu_Min_ is the measure of efficacy of the NAM to reduce an EC_80_ concentration of glutamate.

These data highlight the importance of the amide tail (R group). In general, aliphatic groups (**26f** and **26g**: hmGlu_5_ IC_50_ > 10 μM) as well as substituted phenyl groups (**26k** and **26l**: hmGlu_5_ IC_50_ = inactive) were not tolerated. Expanding the methyl group of thiazole **30a** (hmGlu_5_ IC_50_ = 296 nM) to a nitrile (**26a**) or a cyclobutyl group (**26b**) also led to a complete loss in activity. Similarly, tying the methyl group back onto the thiazole in a fused-ring system (**26c**: hmGlu_5_ IC_50_ > 10 μM) also lead to a drastic decrease in potency. Additionally, pyridines with substitutions *ortho* to the amide linkage proved detrimental to potency (**26m**: hmGlu_5_ IC_50_ = 3.6 μM; **26n**, **26q**, **26s**, **26t:** hmGlu_5_ IC_50_ > 10 μM; **26r**: hmGlu_5_ IC_50_ = inactive). Conversely, 2-pyridyl amides lacking substitution *ortho* to the amide linkage proved to be some of the most potent compounds (hmGlu_5_ IC_50_ < 200 nM). The highly active and unsubstituted pyridine **26p** (hmGlu_5_ IC_50_ = 76 nM) retained potency when substituted with fluorine at the 4-position (**26x**: hmGlu_5_ IC_50_ = 63 nM) versus a 2.5-fold loss in potency when substituted with a methyl group at the 3-position (**26o**: hmGlu_5_ IC_50_ = 192 nM). Interestingly, pyrimidine **26u** afforded a highly potent compound (hmGlu_5_ IC_50_ = 53 nM); however, due to the AO/XO metabolism observed on the pyrimidine ring of **VU0424238** (**10**), we were skeptical **26u** would not pose similar metabolic and toxicity complications.

As extensive efforts were underway to evaluate the thieno­[3,2-*b*]­pyridine core scaffold, a tandem exercise was underway to explore alternative heteroaryl bicyclic cores. For comparison, we maintained the R^2^ group as the 4-methylpyridine. Moreover, we elected to evaluate the new cores utilizing two of the most potent amide R groups (i.e., 4-methylthiazole and 5-fluoropyridine). To begin, commercially available dihalides **31a**–**d** underwent Suzuki cross-couplings to afford intermediates **32** which were subjected to standard palladium-catalyzed carbonylation conditions to give methyl esters **33** (Scheme 3, Route 1). Alternatively, commercially available methyl ester **35** could undergo Suzuki cross-coupling to afford ester **33** (Scheme 3, Route 2). Following basic hydrolysis and subsequent *in situ* generation of the acid chloride, amines were introduced into the reaction to provide analogs **34a**–**e**.

**Scheme 3:**
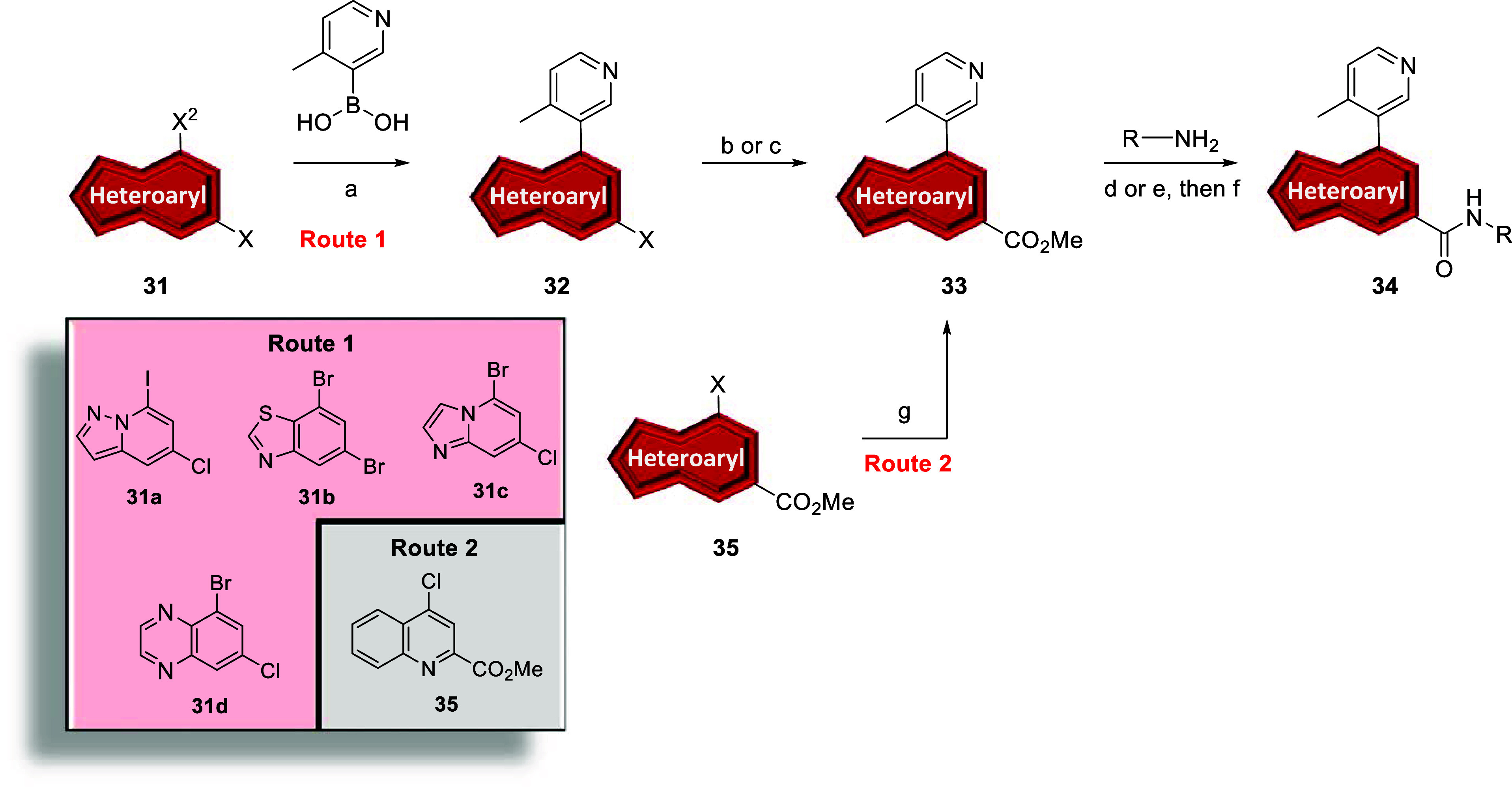
Synthesis of mGlu_5_ NAM Analogs **34** ^a^

a Reagents and conditions: (a) Pd­(dppf)­Cl_2_, Cs_2_CO_3_, 1,4-dioxanes, 90–100 °C, 27–98%; (b) Pd­(OAc)_2_, dppp, TEA, CO_(g)_, MeOH/DMSO (2:1), 80 °C, 27–93%; (c) Pd­(dppf)­Cl_2_, KOAc, MeOH/DMF (1:1), 80 °C, 18 h, 68–83%; (d) NaOH_(aq)_, 1,4-dioxanes, 50 °C, 2–18 h, 98%; (e) Ba­(OH)_2_, THF/H_2_O­(2:1), 50 °C 0.25 h, 98%; (f) amine, POCl_3_, pyridine, 0 °C to rt, 7–41%; (g) Pd­(dppf)­Cl_2_, Cs_2_CO_3_, 1,4-dioxanes, 90 °C, 60%.

In an analogous manner, commercially available dihalides **36a**–**c** underwent Suzuki cross-couplings to afford intermediates **37** which were subjected to standard palladium-catalyzed cyanation conditions to give nitriles **38** in moderate to good yields (Scheme 4, Route 1). Dihalide **36d** first underwent standard palladium-catalyzed cyanation conditions to give nitrile **40**, followed by Suzuki cross-coupling to provide intermediate **38** in moderate yield (Scheme 4, Route 2). Following basic hydrolysis and subsequent *in situ* generation of the acid chloride, amines were introduced into the reaction to provide analogs **39a**–**d**. Analogs **26y-26ac, 34a-e,** and **39a**–**d** were screened against hmGlu_5_ to determine potency with results highlighted in Table 3.

**Scheme 4:**
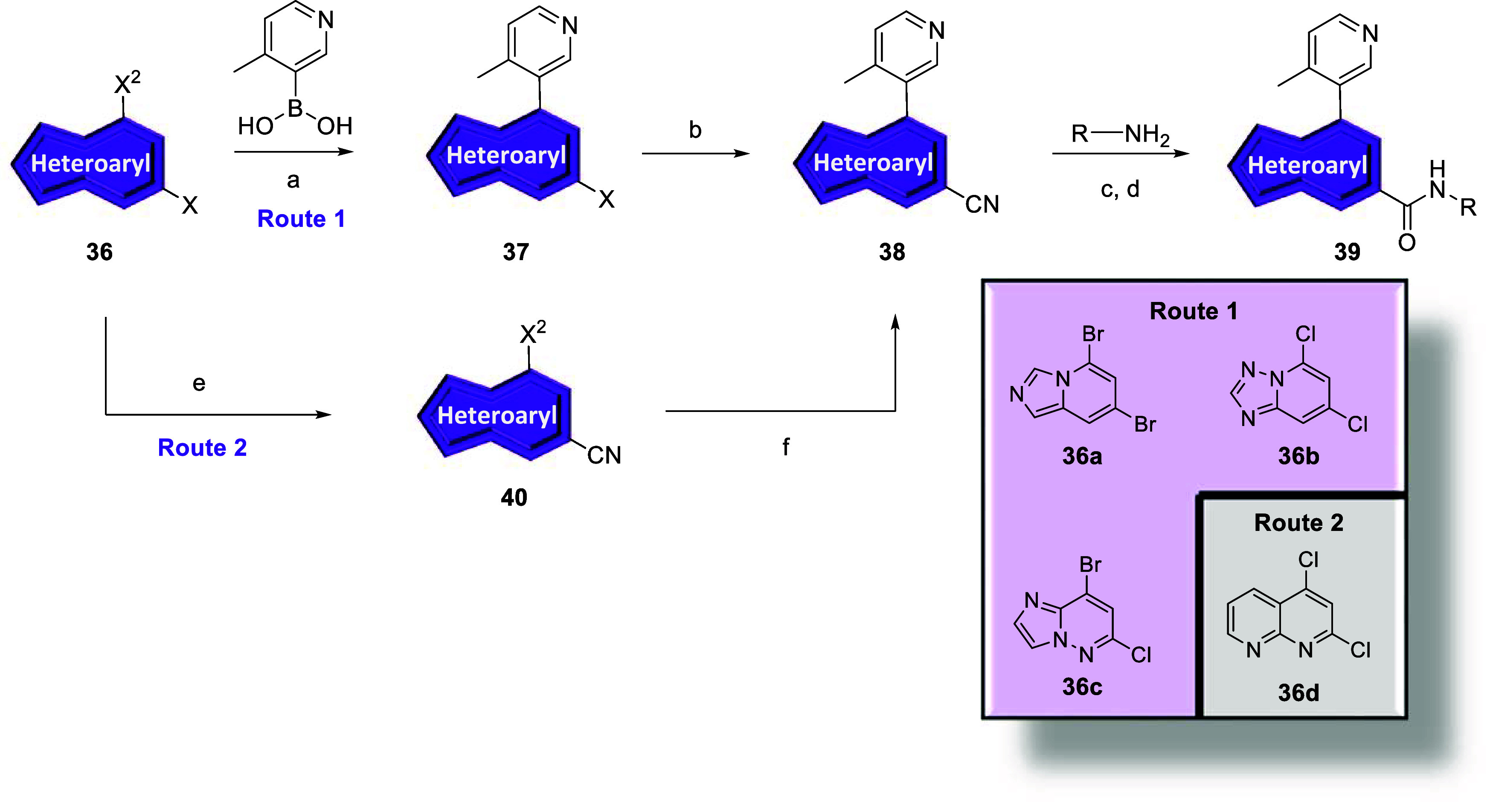
Synthesis of mGlu_5_ Analogs **39** ^a^

a Reagents and conditions: (a) Pd­(dppf)­Cl_2_, Cs_2_CO_3_, 1,4-dioxanes, 90 °C, 3–18 h, 54–87%; (b) Zn­(CN)_2_, Pd­(PPh_3_)_4_, DMF, microwave irradiation 140 °C, 0.25–1 h, 40–54%; (c) NaOH_(aq)_, 1,4-dioxanes, 100 °C, 1–2 h, 98%; (d) amine, POCl_3_, pyridine, 0 °C to rt, 1–32%; (e) Zn­(CN)_2_, Pd­(PPh_3_)_4_, DMF, 120 °C, 2 h, 20%; (f) Pd­(dppf)­Cl_2_, Cs_2_CO_3_, 1,4-dioxanes, 100 °C, 1 h, 30%.

**Table 3:**
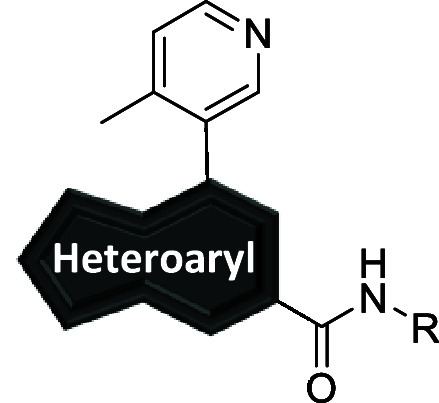

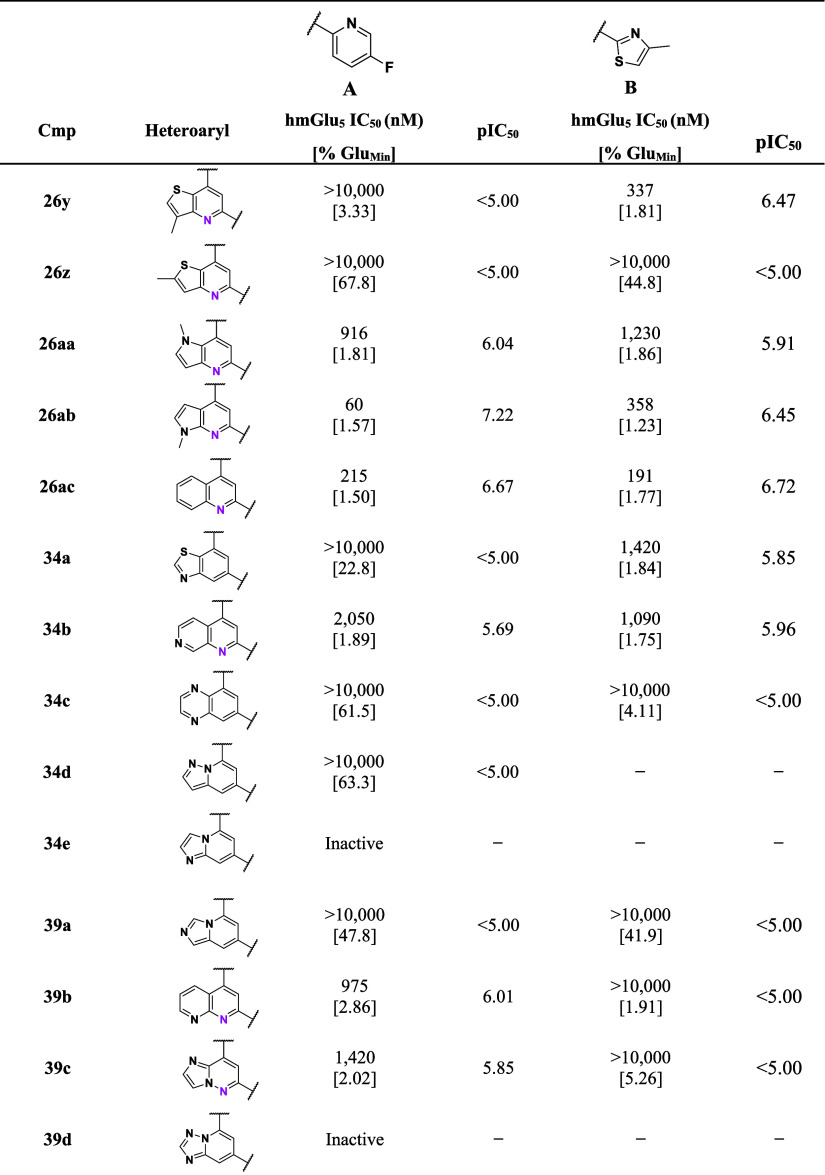
Structures and Activities for Analogs **26y–26ac**, **34**, and **39** ^a^

a Calcium mobilization assays in human mGlu_5_-HEK293A cells were performed in the presence of an EC_80_ fixed concentration of glutamate, *n* = 2 independent experiments in triplicate. The % Glu_Min_ is the measure of efficacy of the NAM to reduce an EC_80_ concentration of glutamate.

A methyl substitution of the thieno­[3,2-*b*]­pyridine at the 2-position (**26y**) lead to a > 158-fold decrease in activity in relation to the 4-fluoropyridine amide tail (**26yA**: hmGlu_5_ IC_50_ > 10 μM). This trend was less pronounced in the context of the 4-methylthiazole amide tail (**26yB**: hmGlu_5_ IC_50_ = 337 nM). On the other hand, a methyl substitution of the thieno­[3,2-*b*]­pyridine at the 3-position lead to a drastic loss of activity (**26zA** and **26zB**: hmGlu5 IC_50_ > 10 μM). Replacement of the core with a benzo­[*d*]­thiazole also resulted in a decrease in potency (**34aA**: hmGlu_5_ IC_50_ > 10 μM; **34aB**: hmGlu_5_ IC_50_ = 1.4 μM) when compared to the original thieno­[3,2-*b*]­pyridine core (**26x**: hmGlu_5_ IC_50_ = 63 nM; **30a**: hmGlu_5_ IC_50_ = 296 nM). Substitution of the core with 1-methyl-1*H*-pyrrolo­[3,2-*b*]­pyridine afforded analogs with a 4–15-fold decrease in potency (**26aa**: hmGlu_5_ IC_50_ ∼ 1 μM) while 1-methyl-1*H*-pyrrolo­[2,3-*b*]­pyridine afforded a highly potent compound (**26abA**: hmGlu_5_ IC_50_ = 60 nM) analogous to the original thieno­[3,2-*b*]­pyridine derivative (**26x**: hmGlu_5_ IC_50_ = 63 nM). Likewise, when the amide tail was exchanged for the 4- methylthiazole (**26abB**: hmGlu_5_ IC_50_ = 358 nM), we observed activity similar to the corresponding thieno­[3,2-*b*]­pyridine analog (**30a**: hmGlu_5_ IC_50_ = 296 nM).

Interestingly, quinoline as a core replacement provided potent analogs without amide tail discrimination (**26acA**: hmGlu_5_ IC_50_ = 215 nM; **26acB**: hmGlu_5_ IC_50_ = 191 nM). Comparatively, substituting with a 1,7-naphthyridine core resulted in a nearly 33-fold and 4-fold decrease in activity in regard to the 4-fluoropyridine tail (**34bA**, hmGlu_5_ IC_50_ = 2.05 μM) and 4-methylthiazole amide tail (**34bB**: hmGlu_5_ IC_50_ = 1.09 μM), respectively. Exchanging the core for a 1,8-naphthyridine led to a 15.5-fold (**39bA**: hmGlu_5_ IC_50_ = 975 nM) and 34-fold (**39bB**: hmGlu_5_ IC_50_ > 10 μM) decrease in activity versus the quinoline core (**26ac**). Several other various 5,6- and 6,6-fused bicyclic heteroaromatic scaffolds were evaluated and demonstrated significant loss of potency (**34cA**, **34dA**, **39aA**, **39aB**: hmGlu_5_ > 10 μM; **34eA**, **39dA**: hmGlu_5_ = inactive).

To determine which compounds would be advanced into extensive in vitro and in vivo drug metabolism and pharmacokinetic (DMPK) characterization, we initially evaluated both human predicted hepatic clearance (CL_hep_) as well rat plasma protein binding (*f*_u,plasma_) of our most potent analogs (hmGlu_5_ IC_50_ ≤ 85 nM) as a method to quickly triage compounds with results highlighted in Table 4. Many of the analogs displayed high human predicted CL_hep_ based on microsomal data (>15 mL/min/kg); however, analogs **30m**, **26u**, and **26abA** were predicted to have moderate human CL_hep_ (12.7–14.2 mL/min/kg) while analog **26x** was predicted to have low human CL_hep_ (4.9 mL/min/kg). Of these four compounds, **26u** was determined to be unstable in rat plasma while **30m** was highly bound to rat plasma proteins resulting in a low fraction unbound (*f*_u,plasma_ = 0.007). Conversely, analogs **26x** and **26abA** were determined to have moderate plasma protein binding (*f*_u,plasma=_ 0.019). Thus, analogs **26x** (**VU6035386**) and **26abA** (**VU6035474**) were selected to advance into selectivity screening as well as an array of DMPK assays including our standard rat plasma:brain level (PBL) cassette paradigm (Table 5).

**Table 4:** In Vitro Predicted Human Hepatic Clearance (CL_hep_) and Rat Plasma Protein Binding (*f*_u,plasma_) of the Most Potent mGlu_5_ NAMs

Cmpd	hmGlu_5_ IC_50_ (nM)	human CL_hep_ (mL/min/kg)	rat *f*_u,plasma_
**26p**	76	16.6	
**26u**	53	13.5	^a^
**26x**	63	4.9	0.019
**26abA**	60	12.7	0.019
**30b**	15	19.3	
**30c**	46	19.7	
**30g**	62	18.5	
**30h**	31	19.0	
**30i**	85	19.4	
**30k**	44	18.6	
**30m**	45	14.2	0.007
**30p**	25	17.7	
**30t**	12	18.4	
**30v**	64	17.0	

a Possibly unstable in rat plasma.

**Table 5:** In Vitro and In Vivo DMPK and Rat PBL Data for Analogs **26x** and **26abA**

	26x	26abA
property	VU6035386	VU6035474
MW	364.4	361.38
xLogP	3.20	2.87
TPSA	67.8	72.7
hmGlu_5_ IC_50_ (nM)	63	60
rmGlu_5_ IC_50_ (nM)	50	159
rmGlu_1,2,4,7,8_ IC_50_ (μM)	inactive	inactive
rmGlu_3_ IC_50_ (μM)	inactive	>10 μM
**in vitro PK parameters** ^a^		
CL_int_ (mL/min/kg), rat	408	439
CL_hep_ (mL/min/kg), rat	60	60
CL_int_ (mL/min/kg), human	6	32
CL_hep_ (mL/min/kg), human	5	12.7
rat *f*_u,plasma_	0.019	0.019
human *f*_u,plasma_	0.011	0.010
rat *f*_u,brain_	0.002	0.002
**brain distribution (0.25 h) (SD rat; 0.2 mg/kg IV)** ^b^ ^,^ ^c^		
*K*_p,brain:plasma_	10.6	5.43
*K*_p,uu,brain:plasma_	1.11	0.57
**rat in vivo PK (IV cassette, 0.2 mg/kg)**		
*t*_1/2_ (min)	39.2	66.2
CL_p_ (mL/min/kg)	198	150
*V*_ss_ (L/kg)	7.13	8.82
**CYP**_**450**_ **IC**_**50**_ **(μM)**		
**1A2**	4.85	1.79
**2C9**	>30	>30
**2D6**	>30	>30
**3A4**	18.2	15.8

a *f*_u_ = Fraction unbound; equilibrium dialysis assay; brain = rat brain homogenates.

b *K*_p_ = total brain to total plasma ratio.

c *K*_p,uu_ = unbound brain (brain *f*_u_ × total brain) to unbound plasma (plasma *f*_u_ × total plasma) ratio.

In regard to physicochemical properties, both **VU6035386** and **VU6035474** possessed molecular weights less than 450 Da with attractive CNS xLogP values (2.87–3.2).^46,47^ Minimal species discrepancy between rat and human potency was observed with both compounds as well as high subtype selectivity when counter screened against other mGlu receptors (Table 5). Both compounds displayed high rat predicted CL_hep_ (rat CL_hep_s > 46 mL/min/kg) which was confirmed in an in vivo IV/PK experiment. In addition to having moderate rat plasma protein binding, both compounds screened had moderate (*f*_u,plasma_s ∼ 0.01) binding to human plasma proteins and were highly bound to rat brain homogenates (*f*_u,brain_ < 0.01). Analog **26x** (rat brain:plasma *K*_p_ = 10.56, *K*_p,uu_ = 1.11) proved to have high CNS distribution of unbound drug while analog **26abA** displayed moderate distribution of unbound drug (rat brain:plasma *K*_p_ = 5.43 *K*_p,uu_ = 0.57). Analog **26x** demonstrated acceptable CYP_450_ profiles against CYP2C9, CYP2D6, and CYP3A4 (IC_50_s ≥ 18.2 μM, > 288-fold selectivity) as well as CYP1A2 (IC_50_ = 4.85 μM, ∼77-fold selectivity). Likewise, **26abA** demonstrated acceptable CYP_450_ profiles against CYP2C9, CYP2D6, and CYP3A4 (IC_50_s ≥ 15.8 μM, >263-fold selectivity); however, **26abA** was less selective in regard to CYP1A2 (IC_50_ = 1.79 μM, ∼ 30-fold selectivity) when compared to **26x**.

## Conclusions

In summary, a scaffold hopping exercise proved to be a successful strategy in generating novel mGlu_5_ NAM chemotypes devoid of the classical aryl/heterobiaryl acetylene moiety associated with metabolic liabilities and hepatotoxicity of historic mGlu_5_ NAMs. Utilizing the *N*-(4-methylthiazol-2-yl)­thieno­[3,2-*b*]­pyridine-5-carboxamide motif of **11** (**VU6031545**), we elected to replace the ether-linked tetrahydrofuran with carbon-linked biaryl/heteroaryl groups which afforded several highly potent mGlu_5_ NAMs (IC_50_s < 100 nM). In a simultaneous effort, an amide screen was performed using various commercially available amines in the presence of the 7-(4-methylpyridin-3-yl)­thieno­[3,2-*b*]­pyridine motif as the core. While this exercise revealed steep SAR trends around the amide moiety, exchanging 4-methylthiazol-2-amine (**30a**) with 5-fluoropyridin-2-amine (**26x**) resulted in a nearly 4-fold increase in potency. Finally, we explored replacing the thieno­[3,2-*b*]­pyridine core with various 5,6- and 6,6-heteroaryl cores. This endeavor identified 1-methyl-1*H*-pyrrolo­[2,3-*b*]­pyridine as a highly potent core in addition to several other moderately potent cores. Although many of these potent analogs displayed high predicted human CL_hep_ (≥15 mL/min/kg) and/or high rat plasma protein binding (*f*_u,plasma_ < 0.01), two analogs (**26x** and **26abA**) displayed moderate fraction unbound in rat plasma (*f*_u,plasma_ = 0.019) and low (**26x**: h CL_hep_ = 5 mL/min/kg) to moderate (**26abA**: h CL_hep_ = 12.7 mL/min/kg) predicted human CL_hep_ which was an improvement over predecessor compound **11** (human CL_hep_ = 18 mL/min/kg). Even though this exercise did not provide mGlu_5_ NAMs with suitable DMPK profiles to warrant further advancement, it did highlight SAR insights for future scaffold designs. These refinements will be reported in due course.

## Methods

### General Information^48^

All chemicals were purchased from commercial vendors and used without further purification. All NMR spectra were recorded on a 400 MHz AMX Bruker NMR spectrometer. ^1^H and ^13^C chemical shifts are reported in δ values in ppm downfield with the deuterated solvent as the internal standard. Low resolution mass spectra were obtained on an Agilent 6120/6150 or Waters QDa (Performance) SQ MS with ESI source. High resolution mass spectra were obtained on an Agilent 6540 UHD Q-TOF with ESI source. Normal phase column chromatography was performed on a Teledyne ISCO CombiFlash R*f*+ system. For compounds that were purified on a Gilson preparative reversed-phase HPLC, the system comprised of a 333 aqueous pump with solvent selection valve, 334 organic pump, GX 271 or GX-281 liquid hander, two column switching valves, and a 155 UV detector. Solvents for extraction, washing and chromatography were HPLC grade. All final compounds were found to be >95% pure by HPLC-MS analysis.

### Synthesis

#### 7-Chlorothieno­[3,2-*b*]­pyridine 4-oxide (**13**)

7-Chlorothieno­[3,2-*b*]­pyridine (2.00 g, 11.8 mmol) was dissolved in DCM (57 mL) and *m*CPBA (3.20 g, 14.1 mmol) was added in portions. The reaction was stirred for 2 h then cooled to 0 °C where an aqueous 10% sodium thiosulfate solution was added. The mixture was added to a separatory funnel and the layers separated. The organic phase was washed with aqueous 10% K_2_CO_3_ and the combined aqueous phases were back extracted with 3:1 CHCl_3_/IPA. The combined organic layers were dried (MgSO_4_), filtered, and concentrated in vacuo. The crude mixture was purified using normal-phase column chromatography on silica gel (0–8% MeOH/DCM with 1% NH_4_OH additive) to afford 2.2 g (98%) of the title compound. ^1^H NMR (400 MHz, CDCl_3_) δ 8.23 (d, *J* = 6.6 Hz, 1H), 7.85 (d, *J* = 5.7 Hz, 1H), 7.73 (d, *J* = 5.7 Hz, 1H), 7.21 (d, *J* = 6.6 Hz, 1H). LRMS: C_7_H_4_ClNOS [M + H]^+^ calc. mass 186.0, found 186.0.

#### 7-Chlorothieno­[3,2-*b*]­pyridine-5-carbonitrile (**17**)

7-Chlorothieno­[3,2-*b*]­pyridine 4-oxide (2.20 g, 11.8 mmol) was dissolved in DCM (60 mL) and trimethylsilyl cyanide (2.96 mL, 23.7 mmol) was added. After 15 min, dimethylcarbamoyl chloride (2.17 mL, 23.7 mmol) was added dropwise and the reaction was stirred at room temperature for 24 h. Additional portions of trimethylsilyl cyanide (2.96 mL, 11.8 mmol) and dimethylcarbamoyl chloride (2.17 mL, 23.7 mmol) were added and the reaction was allowed to stir for another 48 h. The reaction was poured into a separatory funnel and washed with an aqueous 10% K_2_CO_3_ solution. The aqueous phase was back-extracted with DCM and the combined organic layers were dried (MgSO_4_), filtered and concentrated in vacuo. The crude mixture was purified using normal-phase column chromatography on silica gel (0–20% EtOAc/hexanes) to afford 1.89 g (80%) of the title compound. ^1^H NMR (400 MHz, CDCl_3_) δ 7.99 (d, *J* = 5.6 Hz, 1H), 7.67 (d, *J* = 5.6 Hz, 1H), 7.65 (s, 1H). LRMS: C_8_H_3_ClN_2_S [M + H]^+^ calc. mass 195.0, found 195.2.

#### 7-(4-Methylpyridin-3-yl)­thieno­[3,2-*b*]­pyridine-5-carbonitrile (**25**)

7-Chlorothieno­[3,2-*b*]­pyridine-5-carbonitrile (1.00 g, 5.14 mmol), cesium carbonate (5.02 g, 15.4 mmol) and 4-picoline-3-boronic acid (1.41 g, 10.3 mmol) were added to a microwave vial equipped with a stir bar. Next 1,4-dioxane (10 mL) was added to the vial and degassed with nitrogen for 10 min. Next, Pd­(dppf)­Cl_2_ (377 mg, 0.510 mmol) was added and the vial was sealed and irradiated in a microwave at 120 °C for 15 min. The reaction was cooled to room temperature, filtered over Celite, and washed with 10% MeOH/DCM. The organic layers were concentrated and the residue diluted with DCM and dried on Celite. Dry loading and purification using normal-phase chromatography on silica gel (0–2% MeOH/DCM) afforded 1.1 g (81%) of the title compound. ^1^H NMR (400 MHz, CDCl_3_) δ 8.67 (d, *J* = 5.1 Hz, 1H), 8.57 (s, 1H), 7.99 (d, *J* = 5.6 Hz, 1H), 7.75 (d, *J* = 5.6 Hz, 1H), 7.55 (s, 1H), 7.37 (d, 5.2 Hz, 1H), 2.25 (s, 3H). C_14_H_9_N_3_S [M + H]^+^ calc. mass 252.1, found 252.4.

#### 7-(4-Methylpyridin-3-yl)­thieno­[3,2-*b*]­pyridine-5-carboxylic Acid

7-(4-Methylpyridin-3-yl)­thieno­[3,2-*b*]­pyridine-5-carbonitrile (300 mg, 1.20 mmol) was dissolved in 1,4-dioxane (10 mL) then 2*M* aqueous sodium hydroxide (3.0 mL, 6.0 mmol) was added and the reaction was heated at 100 °C for 12 h. The reaction was neutralized to pH 5 with a 2*M* aqueous HCl and the reaction was concentrated in vacuo. The residue was suspended in 10% MeOH/DCM and the salts removed by filtration. The organics were concentrated to afford 322 mg (100%) of the title compound that was used without further purification. ^1^H NMR (400 MHz, DMSO-*d*_6_) δ 8.58 (d, *J* = 5.0 Hz, 1H), 8.50 (s, 1H), 8.13 (d, *J* = 5.5 Hz, 1H), 7.88 (s, 1H), 7.78 (d, *J* = 5.5 Hz, 1H), 7.47 (d, *J* = 5.1 Hz, 1H), 2.15 (s, 3H) (carboxylic acid H not observed). LRMS: C_14_H_10_N_2_O_2_S [M + H]^+^ calc. mass 271.1, found 271.4.

#### N-(5-Fluoropyridin-2-yl)-7-(4-methylpyridin-3-yl)­thieno­[3,2-*b*]­pyridine-5-carboxamide (**26x**, **VU6035386**)

7-(4-Methylpyridin-3-yl)­thieno­[3,2-*b*]­pyridine-5-carboxylic acid (8 mg, 0.03 mmol) and 5-fluoro-2-aminopyridine (4 mg, 0.09 mmol) were dissolved in pyridine (1.5 mL) then cooled to 0 °C. Phosphorus­(V) oxychloride (9 μL, 0.09 mmol) wajjs added and the reaction was allowed to warm to room temperature and stirred 30 min. To the reaction was slowly added water then the mixture concentrated. Crude product was dissolved in DMSO (1 mL) and purified using RP-HPLC (20–55% ACN/0.1% aqueous TFA). Fractions containing product were basified with a saturated aqueous NaHCO_3_ solution and extracted with EtOAc (3x). Solvents were concentrated to give 7 mg (65% yield) of the title compound. ^1^H NMR (400 MHz, CD_3_OD) δ 8.59 (d, *J* = 5.2 Hz, 1H), 8.54 (s, 1H), 8.46 (dd, *J* = 9.1, 4.1 Hz, 1H), 8.31 (d, *J* = 3.0 Hz, 1H), 8.20 (d, *J* = 5.6 Hz, 1H), 8.17 (s, 1H), 7.82 (d, *J* = 5.6 Hz, 1H), 7.72 (ddd, *J* = 9.1, 7.9, 3.0 Hz, 1H), 7.55 (d, *J* = 5.2 Hz, 1H), 2.28 (s, 3H) (NH proton not observed). ^13^C NMR (101 MHz, CD_3_OD) δ 164.18, 158.16 (d, *J* = 250.2 Hz), 156.97, 150.78, 149.28, 148.90, 148.88, 148.81, 147.90, 143.45, 138.28, 136.82 (d, *J* = 26.1 Hz), 135.46, 135.39, 127.36, 126.66 (d, *J* = 19.8 Hz), 126.42, 118.63, 116.15 (d, *J* = 4.6 Hz), 19.44. HRMS: C_19_H_13_FN_4_OS [M + H]^+^ calc. mass 365.0867, found 365.0870.

#### 1-Methyl-4-(4,4,5,5-tetramethyl-1,3,2-dioxaborolan-2-yl)­pyrrolo­[2,3-*b*]­pyridine-6-carbonitrile (**24**)

4-Chloro-1-methyl-1*H*-pyrrolo­[2,3-*b*]­pyridine-6-carbonitrile (678 mg, 3.54 mmol), bis­(pinacolato)­diboron (1.80 g, 7.08 mmol), potassium acetate (1.39 g, 14.2 mmol), Pd­(dppf)­Cl_2_ (260 mg, 0.350 mmol) and 1,4-dioxane (24 mL) were added to a pressure vial. The vial was sealed, evacuated and backfilled with nitrogen (3x). The mixture was stirred for 16 h at 100 °C. After cooling to room temperature, the reaction mixture was filtered through a plug of Celite and washed with EtOAc and DCM. The residue was purified using normal-phase column chromatography on silica gel (0–30% EtOAc/hexanes) to afford 1.0 g (99%) of the title compound. ^1^H NMR (400 MHz, CD_3_OD) δ 7.74 (s, 1H), 7.71 (d, *J* = 3.4 Hz, 1H), 6.92 (d, *J* = 3.4 Hz, 1H), 3.91 (s, 3H), 1.43 (s, 12H). LRMS: C_15_H_18_BN_3_O_2_ [M + H]^+^ calc. mass 284.2, found 284.4.

#### 1-Methyl-4-(4-methyl-3-pyridyl)­pyrrolo­[2,3-*b*]­pyridine-6-carbonitrile (**25**)

3-Bromo-4-methylpyridine (66 mg, 0.39 mmol, 1.1 equiv), 1-methyl-4-(4,4,5,5-tetramethyl-1,3,2-dioxaborolan-2-yl)­pyrrolo­[2,3-*b*]­pyridine-6-carbonitrile (100 mg, 0.350 mmol, 1.00 equiv), Pd­(dppf)­Cl_2_ (39 mg, 0.050 mmol, 0.15 equiv), cesium carbonate (173 mg, 0.530 mmol, 1.50 equiv) and 9:1 acetonitrile:water (1 mL) were added to a pressure vial. The vial was sealed, evacuated and backfilled with nitrogen (3x). After 4 h at 80 °C the reaction mixture was cooled to room temperature and filtered through a plug of Celite washing with EtOAc and DCM. The residue was purified using normal-phase column chromatography on silica gel (0–10% MeOH/DCM) to afford 60 mg (68%) of the title compound. ^1^H NMR (400 MHz, CD_3_OD) δ 8.52 (d, *J* = 5.2 Hz, 1H), 8.45 (s, 1H), 7.74 (d, *J* = 3.5 Hz, 1H), 7.54 (s, 1H), 7.50 (d, *J* = 5.2 Hz, 1H), 6.35 (d, *J* = 3.5 Hz, 1H), 3.98 (s, 3H), 2.27 (s, 3H). LRMS: C_15_H_12_N_4_ [M + H]^+^ calc. mass 249.1, found 249.4.

#### 1-Methyl-4-(4-methyl-3-pyridyl)­pyrrolo­[2,3-*b*]­pyridine-6-carboxylic Acid

1-Methyl-4-(4-methyl-3-pyridyl)­pyrrolo­[2,3-*b*]­pyridine-6-carbonitrile (60 mg, 0.24 mmol, 1.0 equiv) was added to a pressure vial containing 1,4-dioxane (2 mL) and 2*M* aqueous sodium hydroxide (1.2 mL, 2.4 mmol, 10 equiv). The vial was sealed and the mixture was heated overnight at 100 °C. The reaction mixture was first cooled to room temperature then brought to pH 4–5 with 2*M* aqueous HCl. The mixture was concentrated and the residue suspended in 10% MeOH/DCM. Insoluble salts were removed by filtration and the organic solvents concentrated in vacuo. Purification by normal-phase column chromatography on silica gel (0–10% MeOH/DCM) afforded 64 mg (99%) of the title compound. ^1^H NMR (400 MHz, CD_3_OD) δ 8.51 (d, *J* = 5.2 Hz, 1H), 8.46 (s, 1H), 7.87 (s, 1H), 7.67 (d, *J* = 3.5 Hz, 1H), 7.50 (d, *J* = 5.2 Hz, 1H), 6.31 (d, *J* = 3.5 Hz, 1H), 4.03 (s, 3H), 2.27 (s, 3H) (carboxylic acid H not observed). LRMS: C_15_H_13_N_3_O_2_ [M + H]^+^ calc. mass 268.1, found 268.4.

#### N-(5-Fluoropyridin-2-yl)-1-methyl-4-(4-methylpyridin-3-yl)-1H-pyrrolo­[2,3-*b*]­pyridine-6-carboxamide (**26abA**, **VU6035474**)

1-Methyl-4-(4-methyl-3-pyridyl)­pyrrolo­[2,3-*b*]­pyridine-6-carboxylic acid (5 mg, 0.019 mmol) and 5-fluoropyridin-2-amine (2.5 mg, 0.023 mmol) were dissolved in pyridine (1 mL). Phosphorus­(V) oxychloride (5 μL, 0.057 mmol) was added and the reaction allowed to stir for 30 min after which time water was slowly added and solvents removed in vacuo. Purification via RP-HPLC (5–95% ACN/0.1% aqueous TFA) afforded 3.4 mg (48%) of the title. ^1^H NMR (400 MHz, CD_3_OD) δ 6.98 – 6.87 (m, 3H), 6.75 (d, *J* = 3.0 Hz, 1H), 6.41 (s, 1H), 6.22 – 6.11 (m, 2H), 5.95 (d, *J* = 5.2 Hz, 1H), 4.78 (d, *J* = 3.5 Hz, 1H), 2.53 (s, 3H), 0.73 (s, 3H) (NH proton not observed). ^13^C NMR (101 MHz, CD_3_OD) δ 165.03, 158.04 (d, *J* = 249.7 Hz), 149.81, 149.74, 149.09 (d, *J* = 2.2 Hz), 148.02, 147.84, 143.16, 140.11, 136.63 (d, *J* = 26.2 Hz), 136.03, 135.52, 127.22, 126.80 (d, *J* = 20.0 Hz), 124.35, 116.05 (d, *J* = 4.6 Hz), 115.79, 100.07, 31.74, 19.76. HRMS: C_20_H_16_FN_5_O [M + H]^+^ calc. mass 362.1412, found 362.1409.

### Molecular Pharmacology

#### Calcium Mobilization Assays

To measure the functional activity of negative allosteric modulator (NAM) compounds in a cellular assay, human or rat metabotropic glutamate receptor subtype 5 (mGlu_5_) was stably expressed in Human Embryonic Kidney (HEK293A) cells to evoke a decrease in intracellular calcium to an EC_80_ concentration of glutamate (Glu) agonist. Stably expressing mGlu_5_-HEK293A cells were cultured in DMEM medium containing 10% fetal bovine serum, 20 mM HEPES, 2 mM glutamine, 1 mM sodium pyruvate, nonessential amino acid mixture, 0.5 mg/mL G418, and antibiotics/antimycotic. All reagents used were from Life Technologies (Carlsbad, CA) unless otherwise noted.

Briefly, the day before the assay, HEK293A cells stably expressing mGlu_5_ (20,000 cells/20 μL/well) were plated in in black-walled, clear-bottomed, amine-treated 384 well plates (Corning) in the assay medium (DMEM containing 10% dialyzed fetal bovine serum, 20 mM HEPES, 1 mM sodium pyruvate, and antibiotics/antimycotic). Cells were incubated overnight at 37 °C in the presence of 5% CO_2_. The next day, calcium assay buffer (Hank’s balanced salt solution (HBSS), 20 mM HEPES, 2.5 mM Probenecid, 4.16 mM sodium bicarbonate (Sigma-Aldrich, St. Louis, MO)) was prepared to dilute compounds, agonists, and Fluo-4-acetomethoxyester (Fluo-4-AM, Ion Biosciences), fluorescent calcium indicator dye. Compounds were serially diluted 1:5 into 10-point concentration response curves in DMSO using a Bravo Liquid Handler (Agilent, Santa Clara, CA), transferred to a 384 well daughter plates using an Echo acoustic liquid handler (Beckman Coulter, Indianapolis, Indiana), and diluted in assay buffer to a 2X final concentration. The agonist plates were prepared using glutamate (Tocris) concentrations for the EC_20_, EC_80_ and EC_Max_ responses by diluting in assay buffer to a 5X final concentration. A 2X dye solution (2.3 μM) was prepared by mixing a 2.3 mM Fluo-4-AM stock in DMSO with 10% (w/v) pluronic acid F-127 in a 1:1 ratio in assay buffer. Using a microplate washer (BioTek, Winooski, VT), cells were washed with assay buffer 3 times to remove media. After the final wash, 20 μL of assay buffer remained in the cell plates. Immediately, 20 μL of the 2X dye solution (final 1.15 μM) was added to each well of the cell plate using a Multidrop Combi dispenser (Thermo Fisher, Waltham, MA). After cells were incubated with the dye solutions for 45 min at 37 °C in the presence of 5% CO_2_, the dye solutions were removed and replaced with assay buffer using a microplate washer, leaving 20 μL of assay buffer in the cell plate. The compound, agonist, and cell plates were placed inside the Functional Drug Screening System (FDSS 7000 or μCell kinetic imaging plate reader, Hamamatsu, Japan) to measure the calcium flux. Assays were run at 37 °C. A triple add protocol was used to measure Ca kinetics. Briefly, after establishment of a fluorescence baseline for 2 s (excitation, 480 nm; emission, 530 nm), 20 μL of test compound was added to the cells and the response was measured for 142 s. This was followed by the addition of 10 μL (5X) of an EC_20_ concentration of Glu agonist, and the response of the cells was measured for 125 s. A third addition occurred by adding 12 uL (5X) of an EC_80_ concentration of Glu agonist and the response of the cells was measured for 90 s. Vehicle (0.6% DMSO) in assay buffer was added to the control wells at the first add for measuring glutamate EC_20_, EC_80_, and EC_Max_ responses. Calcium fluorescence was recorded as fold over basal fluorescence and raw data were normalized to the maximal response to Glu agonist (EC_Max_). Compound-evoked decreases in calcium response in the presence of Glu EC_80_ agonist were determined as inhibition activity, and potency (IC_50_) and maximum inhibition responses (% Glu_Min_) of compounds were determined using a four-parameter logistical equation using GraphPad Prism (La Jolla, CA) or the Dotmatics software platform (Woburn, MA):

$$y=\mathrm{bottom}+\frac{\mathrm{top}-\mathrm{bottom}}{1+10^{(\log\mathrm{EC}50-A)\mathrm{Hillslope}}}$$

where *A* is the molar concentration of the compound; bottom and top denote the lower and upper plateaus of the concentration–response curve; HillSlope is the Hill coefficient that describes the steepness of the curve; and IC_50_ is the molar concentration of compound required to generate a response halfway between the top and bottom.

## Supplementary Material



## Notes

General methods for the synthesis and characterization for key compounds, experimental details for all assays performed (calcium mobilization; in vitro and in vivo DMPK) (PDF)
