# Supplementary material for: Discovery of 7‑(Pyridin-3-yl)thieno[3,2‑b]pyridine-5-carboxamides as Negative Allosteric Modulators of Metabotropic Glutamate Receptor Subtype 5
Source: ACS Chem Neurosci. 2026 Jan 26;17(3):610–23. doi: 10.1021/acschemneuro.5c00896 (PMC12879744; doi:10.1021/acschemneuro.5c00896)
Supplement: Supplementary file 1 [file cn5c00896_si_001.pdf]

Supplemental Information for

Discovery of 7-(Pyridin-3-yl)thieno[3,2-*b*]pyridine-5-carboxamides as Negative Allosteric Modulators of Metabotropic Glutamate Receptor Subtype 5

Scott H. Henderson[a,b], Anna E. Ringuette[a,b], David L. Whomble[a,b,h], Rory A. Capstick[a,b,h], Alexa E. Richardson[a,b], Mallory A. Maurer[a,b], Natasha B. Billard[a,b], Xia Lei[a,b,h], Joshua C. Wilkinson[a,b,h], Sri H. Kethanapalli[a,b,h], Hyekyung P. Cho[a,b,h], Alice L. Rodriguez[a,b,h], Colleen M. Niswender[a,b,d,e,f,g,h], Weimin Peng[b], Jerri M. Rook[b], Sichen Chang[a,b,h], Anna L. Blobaum[a,b], Olivier Boutaud[a,b,h], Andrew S. Felts[a,b], P. Jeffrey Conn[a,b,f], Craig W. Lindsley[a,b,c,d,g,h*], Kayla J. Temple[a,b,h *]

[a]Warren Center for Neuroscience Drug Discovery, Vanderbilt University, Nashville, TN 37232, USA
[b]Department of Pharmacology, Vanderbilt University School of Medicine, Nashville, TN 37232, USA
[c]Department of Chemistry, Vanderbilt University, Nashville, TN 37232, USA
[d]Department of Biochemistry, Vanderbilt University, Nashville, TN 37232, USA
[e]Vanderbilt Kennedy Center, Vanderbilt University Medical Center, Nashville, TN 37232, USA
[f]Vanderbilt Brain Institute, Vanderbilt University School of Medicine, Nashville, TN 37232, USA
[g]Vanderbilt Institute of Chemical Biology, Vanderbilt University School of Medicine, Nashville, TN 37232, USA
[h]Vanderbilt Institute for Therapeutic Advances, Vanderbilt University, Nashville, TN 37232, USA

*Corresponding authors' email:

kayla.temple@vanderbilt.edu
craig.lindsley@vanderbilt.edu

Table of Contents

General Instrumentation Methods S2
Molecular Pharmacology Methods S3
mGlu Receptor Selectivity Screening Assay S3
DMPK Methods S6
IV plasma-brain level determination (PBL). S6
Binding in plasma from rat and human. S7
Binding in brain homogenates from rat. S8
Intrinsic Clearance in Rat and Human Liver Microsomes S8
LC-MS/MS Analysis S9
HR-MS Spectra of Key Compounds S11
^{1}H and ^{13}C NMR Spectra of Key Compounds S15

General Instrumentation Methods.

All reactions were carried out employing standard chemical techniques. Solvents used for extraction, washing, and chromatography were HPLC grade. All reagents were purchased from commercial sources and were used without further purification.

Automated flash column chromatography was performed on a Biotage Isolera 1 or a Teledyne ISCO CombiFlash system. RP-HPLC was performed on a Gilson preparative reversed-phase HPLC system comprised of a 333 aqueous pump with solvent-selection valve, 334 organic pump, GX-271 or GX-281 liquid hander, two column switching valves, and a 155 UV detector. Absorbance was typically monitored at 215 or 220 nm. Column: Phenomenex Axia-packed Gemini C18, 5 µm. Mobile phase: CH_3CN in H_2O (0.1% TFA) or CH_3CN in H_2O (0.05% *v/v* NH_4OH) under the specified gradient, then hold 95% CH_3CN in 5% aqueous phase, 50 mL/min, 23° C. All compounds were found to be >95% pure by LCMS analysis.

All NMR spectra were recorded on a 400 MHz AMX Bruker NMR spectrometer. ^1H and ^{13}C chemical shifts are reported in δ values in ppm downfield with the deuterated solvent as the internal standard. Data are reported as follows: chemical shift, multiplicity (s = singlet, d = doublet, t = triplet, q = quartet, b = broad, m = multiplet), integration, coupling constant (Hz).

Low resolution mass spectra (LRMS) were obtained on an Agilent 6120/6150 or Waters QDa (Performance) SQ MS with ESI source. *Method A (Agilent 6120/6150):* MS parameters were as follows: fragmentor: 70, capillary voltage: 3000 V, nebulizer pressure: 30 psig, drying gas flow: 13 L/min, drying gas temperature: 350 ºC. Samples were introduced via an Agilent 1290 UHPLC comprised of a G4220A binary pump, G4226A ALS, G1316C TCC, and G4212A DAD with ULD flow cell. UV absorption was generally observed at 215 nm and 254 nm with a 4 nm bandwidth. Column: Waters Acquity BEH C18, 1.0 x 50 mm, 1.7 um. Gradient conditions: 5% to 95% CH_3CN in H_2O (0.1% TFA) over 1.4 min, hold at 95% CH_3CN for 0.1 min, 0.5 mL/min, 55 ºC. *Method B (Agilent 6120/6150):* MS parameters were as follows: fragmentor: 100, capillary voltage: 3000 V, nebulizer pressure: 40 psig, drying gas flow: 11 L/min, drying gas temperature: 350 ºC. Samples were introduced via an Agilent 1200 HPLC comprised of a degasser, G1312A binary pump, G1367B HP-ALS, G1316A TCC, G1315D DAD, and a Varian 380 ELSD (if applicable). UV absorption was generally observed at 215 nm and 254 nm with a 4 nm bandwidth. Column: Thermo Accucore C18, 2.1 x 30 mm, 2.6 um. Gradient conditions: 7% to 95% CH_3CN in H_2O (0.1% TFA) over 1.6 min, hold at 95% CH_3CN for 0.35 min, 1.5 mL/min, 45 ºC. *Method C (Waters QDa (Performance) SQ MS)*: MS parameters were as follows: cone voltage: 15 V, capillary voltage: 0.8 kV, probe temperature: 600° C. Samples were introduced via an Acquity I-Class PLUS UPLC comprised of a BSM, FL-SM, CH-A, and PDA. UV absorption was generally observed at 215 nm and 254 nm; 4 nm bandwidth. Column: Phenomenex EVO C18, 1.0

x 50 mm, 1.7 um. Column temperature: 55° C. Flow rate: 0.4 mL/min. Default gradient: 5% to 95% CH_3CN (0.05% TFA) in H_2O (0.05% TFA) over 1.4 min (curve 6), hold at 95% CH_3CN for 0.1 min. "Polar" (2% to 70% CH_3CN (0.05% TFA) in H_2O (0.05% TFA) over 0.8 min (curve 6), transition to 95% CH_3CN over 0.1 min (curve 6), hold at 95% CH_3CN for 0.6 min.) and "Non-Polar" (40% to 95% CH_3CN (0.05% TFA) in H_2O (0.05% TFA) over 1.4 min (curve 6), hold at 95% CH_3CN for 0.1 min.) gradients were also available. *Method D (Waters QDa (Performance) SQ MS):* MS parameters were as follows: cone voltage: 15 V, capillary voltage: 0.8 kV, probe temperature: 600° C. Samples were introduced via an Acquity I-Class PLUS UPLC comprised of a BSM, FL-SM, CH-A, and PDA. UV absorption was generally observed at 215 nm and 254 nm with a 4 nm bandwidth. Column: Phenomenex EVO C18, 1.0 x 50 mm, 1.7 um. Column temperature: 55° C. Flow rate: 0.4 mL/min. Default gradient: 5% to 95% CH_3CN in H_2O (5 mM NH_4HCO_3) over 1.4 min (curve 6), hold at 95% CH_3CN for 0.1 min. "Polar" (2% to 70% CH_3CN in H_2O (5 mM NH_4HCO_3) over 0.8 min (curve 6), transition to 95% CH_3CN over 0.1 min (curve 6), hold at 95% CH_3CN for 0.6 min.) and "Non-Polar" (40% to 95% CH_3CN in H_2O (5 mM NH_4HCO_3) over 1.4 min (curve 6), hold at 95% CH_3CN for 0.1 min.) gradients were also available.

High resolution mass spectra (HRMS) were obtained on an Agilent 6540 UHD Q-TOF with ESI source. MS parameters were as follows: fragmentor: 150, capillary voltage: 3500 V, nebulizer pressure: 60 psig, drying gas flow: 13 L/min, drying gas temperature: 275 ºC. Samples were introduced via an Agilent 1200 UHPLC comprised of a G4220A binary pump, G4226A 3 ALS, G1316C TCC, and G4212A DAD with ULD flow cell. UV absorption was observed at 215 nm and 254 nm with a 4 nm bandwidth. Column: Agilent Zorbax Extend C18, 1.8 µm, 2.1 x 50 mm. Gradient conditions: 5% to 95% CH_3CN in H_2O (0.1% formic acid) over 1 min, hold at 95% CH_3CN for 0.1 min, 0.5 mL/min, 40 ºC.

Safety statement*: no unexpected or unusually high safety hazards were encountered.*

Molecular Pharmacology Methods.

mGlu Receptor Selectivity Screening Assay

The mGlu isoform selectivity screening of mGlu5 NAM compounds at rat $mGlu_1$, $mGlu_7$ or $mGlu_8$ was performed in calcium mobilization assay. Tetracycline-inducible rat $mGlu_1$ stably expressed in T-REx^{TM}-293 cells (Life Technologies) were cultured in Dulbecco's Modified Eagle Media (DMEM) containing 10% tetracycline-negative fetal bovine serum (GeminiBio), 20 mM HEPES, 1 mM sodium pyruvate, 2 mM L-glutamine, non-essential amino acids mixture, antibiotics/antimycotic, 100 µg/mL hygromycin, and 5 µg/mL blasticidin. Human Embryonic

Kidney 293 (HEK293 cells) stably co-expressing rat $mGlu_7$ or $mGlu_8$ and the promiscuous G protein $G_{\alpha15}$ (mouse) were maintained in DMEM containing 10% fetal bovine serum, 20 mM HEPES, 1 mM sodium pyruvate, 2 mM L-glutamine, non-essential amino acids mixture, 100 units/mL penicillin/streptomycin, 700 µg/mL G418 sulfate, and 0.6 µg/mL puromycin. All cell culture reagents were purchased from Invitrogen (Carlsbad, CA).

The day before the assay, rat $mGlu_7$_$G_{\alpha15}$_HEK cells (20,000 cells/20 µL/well) or rat $mGlu_8$_$G_{\alpha15}$_HEK cells (15,000 cells/20 µL/well) were plated in black-walled, clear-bottomed, poly-D-lysine coated, 384 well plates (Corning) in the assay medium (DMEM containing 10% dialyzed FBS, 20 mM HEPES, 1 mM sodium pyruvate, 100 units/mL penicillin/streptomycin. To induce rat $mGlu_1$ expression, the $mGlu_1$-T-REx^{TM}-293 cells were plated in the same assay medium containing 10 ng/ml tetracycline at the seeding density of 20,000 cells/20 µL/well. The cells were grown overnight at 37 °C in the presence of 5% CO_2.

The next day, calcium assay buffer (Hank's balanced salt solution (HBSS), 20 mM HEPES, 2.5 mM Probenecid, 4.16 mM sodium bicarbonate (Sigma-Aldrich, St. Louis, MO)) was prepared to dilute compounds, agonists, and Fluo-4-acetomethoxyester (Fluo-4-AM, Ion Biosciences), fluorescent calcium indicator dye. Compounds were serially diluted 1:3 into 10-point concentration response curves in DMSO using a Bravo Liquid Handler (Agilent, Santa Clara, CA), transferred to a 384 well daughter plates using an Echo acoustic liquid handler (Beckman Coulter, Indianapolis, Indiana), and diluted in assay buffer to a 2X final concentration. The agonist plates were prepared using glutamate (for $mGlu_1$ and $mGlu_8$) or DL-AP4 (for $mGlu_7$) concentrations for the EC_{20}, EC_{80} and EC_{Max} responses by diluting in assay buffer to a 5X final concentration. A 2X dye solution (2.3 µM) was prepared by mixing a 2.3 mM Fluo-4-AM stock in DMSO with 10% (w/v) pluronic acid F-127 in a 1:1 ratio in assay buffer. Using a microplate washer (BioTek, Winooski, VT), cells were washed with assay buffer 3 times to remove media. After the final wash, 20 µL of assay buffer remained in the cell plates. Immediately, 20 µL of the 2X dye solution (final 1.15 µM) was added to each well of the cell plate using a Multidrop Combi dispenser (Thermo Fisher, Waltham, MA). All steps of the assay for $mGlu_7$ and $mGlu_8$ were performed at room temperature and at 37 °C for $mGlu_1$. After cells were incubated with the dye solutions for 45 min in the presence of 5% CO_2, the dye solutions were removed and replaced with assay buffer using a microplate washer, leaving 20 µL of assay buffer in the cell plate. The compound, agonist, and cell plates were placed inside the Functional Drug Screening System (FDSS 7000 or µCell kinetic imaging plate reader, Hamamatsu, Japan) to measure the calcium flux. A triple add protocol was used to measure Ca kinetics. Briefly, after establishment of a fluorescence baseline for 2 seconds (excitation, 480 nm; emission, 530 nm), 20 µL of test compound was added to the cells and the response was measured for 142 seconds. This was followed by the addition of 10 µL (5X) of an EC_{20} concentration of agonist, and the response of the cells was measured for 125 seconds. A third addition occurred by adding 12 uL (5X) of an EC_{80} concentration of agonist and the response of the cells was measured for 90 seconds. Vehicle (0.6 % DMSO) in assay buffer was added to the control wells at the 1st add for measuring agonist EC_{20}, EC_{80}, and EC_{Max} responses. Calcium

fluorescence was recorded as fold over basal fluorescence and raw data were normalized to the maximal response to agonist (glutamate for $mGlu_1$ and $mGlu_8$ or DL-AP4 for $mGlu_7$). Compound-evoked decreases in calcium response in the presence of agonist EC_{80} agonist were determined as inhibition activity, and potency (IC_{50}) and maximum inhibition responses (% Glu_{Min}) of compounds were determined using a four-parameter logistical equation using GraphPad Prism (La Jolla, CA) or the Dotmatics software platform (Woburn, MA) :

$$y = bottom + \frac{top - bottom}{1 + 10^{(LogEC50 - A)Hillslope}}$$

where *A* is the molar concentration of the compound; *bottom* and *top* denote the lower and upper plateaus of the concentration-response curve; Hillslope is the Hill coefficient that describes the steepness of the curve; and EC_{50} is the molar concentration of compound required to generate a response halfway between the *top* and *bottom*.

The mGlu isoform selectivity screening of mGlu5 NAM compounds at rat $mGlu_2$, $mGlu_3$ or $mGlu_4$ was performed in thallium flux assay. Human embryonic kidney 293 (HEK293) cell lines stably co-expressing rat $mGlu_2$, $mGlu_3$, or $mGlu_4$ and G protein-coupled inwardly rectifying potassium (GIRK) channels were maintained in growth medium containing DMEM/F12 containing 10% FBS, 20 mM HEPES, 1 mM sodium pyruvate, 2 mM L-glutamine, non-essential amino acids mixture, 100 units/ml penicillin/streptomycin, 700 µg/mL G418, and 0.6 µg/mL puromycin.

The day before assay, rat $mGlu_{2,}$ $mGlu_3$ or $mGlu_4$-GIRK cells were plated into 384 well, black-walled, clear-bottom poly-D-lysine coated plates at a density of 15,000 cells/20 µL/well in DMEM containing 10% dialyzed FBS, 20 mM HEPES, 1 mM sodium pyruvate, and 100 units/ml penicillin/streptomycin. The cells were incubated overnight at 37 °C in the presence of 5% CO_2.

The next day, GIRK assay buffer (Hank's balanced salt solution (HBSS), 20 mM HEPES, and 4.16 mM sodium bicarbonate, pH 7.4) was prepared to dilute compounds, and Thallos-acetomethoxyester (Thallos-AM, Ion Biosciences), fluorescent thallium sensitive dye. Compounds were serially diluted 1:3 into 10-point concentration response curves in DMSO using a Bravo Liquid Handler (Agilent, Santa Clara, CA), transferred to a 384 well daughter plates using an Echo acoustic liquid handler (Beckman Coulter, Indianapolis, Indiana), and diluted in assay buffer to a 2X final concentration. A 2X dye solution (1.36µM) was prepared by mixing a 2.97 mM Thallos-AM stock in DMSO with 10% (w/v) pluronic acid F-127 in a 1:1 ratio in assay buffer. The agonist plates were prepared using glutamate concentrations for the EC_{80} and EC_{Max} responses to a 5X final concentration in thallium stimulation buffer (125 mM $NaHCO_3$, 1.8 mM $CaSO_4$, 1 mM $MgSO_4$, 5 mM glucose, 12.5 mM Tl_2SO_4, 10 mM HEPES, pH 7.4). Using a microplate washer (BioTek, Winooski, VT), cells were washed with GIRK assay buffer 3 times to remove media. After the final wash, 20 µL of assay buffer remained in the cell plates. Immediately, 20 µL of the

2X dye solution (final 0.68 μM) was added to each well of the cell plate using a Multidrop Combi dispenser (Thermo Fisher, Waltham, MA). After cells were incubated with the dye solutions for 60 min at room temperature, the dye solutions were removed and replaced with GIRK assay buffer using a microplate washer, leaving 20 μL of assay buffer in the cell plate. The compound, agonist, and cell plates were placed inside the Functional Drug Screening System (FDSS 7000 or μCell kinetic imaging plate reader, Hamamatsu, Japan) to measure the thallium flux using a double add protocol. After establishment of a fluorescence baseline (excitation, 480 nm; emission, 530 nm), 20 μL (2x) of test compound was added to the cells at 2 second and the response was measured. 140 seconds later, 10 μL (5x) of an EC_{80} concentration of glutamate or vehicle in thallium stimulus buffer was added to the cells, and the response of the cells was measured for an additional 158 seconds. Multiple reference wells containing no compound (DMSO vehicle) received either no glutamate (for a baseline reference), EC_{80} glutamate, or EC_{Max} glutamate (for normalization to maximum response). Fluorescence was measured throughout the experiment at a frequency of 1 measurement per every 2 seconds before the glutamate/thallium addition and a frequency of 1 measurement per every 1 second after the glutamate/thallium addition. Each compound concentration series occurred one time in each plate, and replicates from two or three plates were used in each experimental run. Thallium solutions and plastic solid waste were handled and disposed of according to guidelines from the Vanderbilt University Chemical Safety department. Data were normalized using a static ratio function (F/F_0) by dividing every fluorescent measurement by the initial fluorescent value for the corresponding well. The increase in signal resulting from the glutamate/thallium addition of the second add was measured by determining the slope from the time window of 145 seconds to 155 seconds. The average of all baseline slopes (no compound, no glutamate) was determined and this value was subtracted from all other slope values. The average of all EC_{Max} slopes was determined, and this value was used to normalize the baseline-corrected slopes to a percent max value ($\%E_{Max}$). Compound-evoked decreases in thallium flux response in the presence of glutamate EC_{80} agonist were determined as inhibition activity, and potency (IC_{50}) and maximum inhibition responses ($\%\ Glu_{Min}$) of compounds were determined using a four-parameter logistical equation as described above.

DMPK Methods

IV plasma-brain level determination (PBL).

In-life phase

Compounds were formulated as a solution in ethanol, PEG400, and DMSO (1:4:5 v/v, respectively) at a concentration of 1 mg/mL and administered as a single 0.2 mg/kg IV dose (0.5 mL/kg) to male, Sprague Dawley rats (n = 1; 342 gram body weights) via injection into a surgically-implanted jugular vein catheter. Blood samples were collected serially from a surgically implanted carotid artery catheter in each animal over multiple post-administration time points (0.033, 0.117, 0.25, 0.5, 1, 2, 4, 7, and 24 hours) into chilled, K2EDTA anticoagulant-

fortified tubes and immediately placed on wet ice. The blood samples were then centrifuged (1700 rcf, 5 minutes, 4 °C) in order to obtain plasma samples, which were stored at -80 ˚C until analysis by LC-MS/MS.

For determination of the brain over plasma ratio (K_p), compounds were formulated in 10% ethanol, 40% PEG400 and 50% DMSO (v/v/v) and administered as a single 0.2 mg/kg IV dose (0.5 mL/kg) to male, Sprague Dawley rats (n = 1; 316 gram body weights) via injection into a surgically-implanted jugular vein catheter. At 15 min post dosing, blood sample was collected serially (i.e., terminally) into chilled, K2EDTA anticoagulant-fortified tube and immediately placed on wet ice. The blood sample was then centrifuged (1700 rcf, 5 minutes, 4 °C) to obtain plasma sample. At the same post-administration time point, whole brain sample was obtained by rapid dissection, rinsed with saline, and immediately frozen in individual tissue collection box (dry ice). All brain and plasma samples were stored at -80 °C until analysis by LC-MS/MS.

Samples preparation for bioanalysis

Plasma samples from the in-life phase of the study were thawed at ambient temperature (benchtop), and then aliquots (20 µL per sample) were transferred to a 96-shallow-well (V-bottom) plate. Matrix-matched quality control (QC) samples and a standard curve of **VU6035386** or **VU6035474** (1 mg/mL DMSO stock solution) were prepared in blank rat plasma (K2EDTA-treated) or blank brain homogenate via serial dilution and transferred (20 µL each) to the plate along with multiple blank plasma and brain homogenate samples. Acetonitrile (120 µL) containing IS (10 nM carbamazepine) was added to each well of the plate to precipitate protein. The plate was then centrifuged (4000 rcf, 5 minutes, ambient temperature), and resulting supernatants (60 µL each) were transferred to a new 96-shallow-well (V-bottom) plate containing an equal volume (60 µL per well) of water (Milli-Q purified). The plate was then sealed in preparation for LC-MS/MS analysis.

Preparation of brain samples was identical to that of plasma samples except for the following modifications. While thawing, brains were weighed (inside their collection boxes using a universal empty collection box tare weight) and then subjected to mechanical homogenization (Mini-BeadBeater™, BioSpec Products, Inc., Bartlesville, OK) in the presence of zirconia/silica beads (1.0 mm) and extraction buffer (isopropanol:water, 7:3, v/v; 3 mL per sample, corrected for post-quantitation). Homogenized brain samples were then centrifuged (4000 rcf, 5 minutes, ambient temperature), and 5 uL of the supernatant was diluted in 15 uL of blank plasma for quantification of the analyte. The plasma standard curve and QCs were used for compounds quantitation in brain.

<u>Binding in plasma from rat and human.</u>

Determination of compounds' fraction unbound (f_u) in plasma from rat and human was conducted *in vitro* via equilibrium dialysis using HTDialysis membrane plates. Dialysis membranes (four paired strips per HTD assay) were hydrated as described by the manufacturer and inserted into the HTD plate, which was assembled and prepared for sample addition by the dispensing of blank buffer (DPBS, 100 µL/well) into the 'top half' of each membrane-split well. Each compound was diluted into plasma from each species (5 µM final concentration), which was

aliquoted in triplicate to the 'bottom half' of the prepared HTD plate wells. The HTD plate was sealed and incubated for 6 hours at 37 °C. Following incubation, each well (both top and bottom halves) were transferred (20 µL) to the corresponding wells of a 96-shallow-well (V-bottom) plate. The daughter plates were then matrix-matched (buffer side wells received equal volume of plasma, and plasma side wells received equal volume of buffer), and extraction solution (120 µL; acetonitrile containing 50 nM carbamazepine as IS) was added to all wells of both daughter plates to precipitate protein and extract test article. The plates were then sealed and centrifuged (3500 rcf) for 10 minutes at ambient temperature. Supernatant (60 µL) from each well of the daughter plates was then transferred to the corresponding wells of new daughter plates (96-shallow-well, V bottom) containing water (Milli-Q, 60 µL/well), and the plates were sealed in preparation for LC-MS/MS analysis (see below).

f_u was calculated as (analyte to IS MS peak area ratio from Trans-buffer side) / (analyte to IS MS peak area ratio from Cis-plasma side). Mean values for each species were calculated from 3 replicates.

<u>Binding in brain homogenates from rat.</u>

Determination of fraction unbound (f_u) in brain homogenate from rat was conducted using the same methodology and procedure than described for plasma protein binding assay with the following modifications: 1) a final compound concentration of 1 µM was used, 2) naïve rat brains were homogenized in DPBS (1:3 composition of brain: DPBS, w/w) using a Mini-Bead Beater™ machine in order to obtain brain homogenate.

The diluted fraction unbound (f_{u2}) in brain was calculated as (analyte to IS MS peak area ratio from Trans-buffer side) / (analyte to IS MS peak area ratio from Cis-brain homogenate side). Undiluted fraction unbound for the brain was calculated using the following equation:

$$f_u = \frac{1/4}{\left\{\left(\frac{1}{f_{u2}}\right) - 1\right\} + 1/4}$$

Mean values for each species were calculated from 3 replicates.

<u>Intrinsic Clearance in Rat and Human Liver Microsomes</u>

The *in vitro* intrinsic clearance (CL_{int}) was investigated in commercially obtained hepatic microsomes from rat and human donors using the substrate depletion (i.e., loss-of-parent vs. time, or $t_{1/2}$ method) approach with analyte detection via liquid chromatography-tandem mass spectrometry (LC-MS/MS). For each species, mean %parent remaining values at each time point were calculated from replicates raw data (analyte:IS peak area ratios) and used to determine *in vitro* $t_{1/2}$ and CL_{int}.

Experiments were carried out using a robot-assisted (TECAN model Evo 200). Compound was incubated (1 µM final concentration) in buffer (100 mM potassium phosphate pH 7.4 with 3 mM $MgCl_2$) containing hepatic microsomes (0.5 mg/mL final concentration) from multiple species, discretely, at 37 °C under constant orbital shaking. After 5 minutes (pre-incubation), reactions were initiated by addition of nicotinamide adenine dinucleotide

phosphate (NADPH, 1 mM final concentration). At selected time intervals (0, 3, 7, 15, 25, and 45 minutes) post-addition of NADPH, aliquots (50 μL) were taken and placed into a 96-shallow-well plate containing ice cold acetonitrile (150 μL) with carbamazepine (IS, 50 nM). The plates were then centrifuged (3000 rcf at 4 °C) for 10 minutes. The supernatants were transferred to a new 96-shallow-well daughter plate and diluted (1:1 v/v) with water (Milli-Q filtered). The plates were then sealed in preparation for LC-MS/MS analysis (see below).

Raw LC-MS/MS peak area data generated from the assay samples were used to construct natural log-transformed %parent remaining vs. time plots (using t = 0 minute post-NADPH addition sample data as starting point set to 100%). *In vitro* compound half-life ($t_{1/2}$) values were obtained using the following equation:

$$t_{1/2} = \frac{Ln(2)}{k}$$

Where k is the slope from linear regression analysis of the natural log-transformed data (using means from all replicates at each time point). Resulting $t_{1/2}$ values were then used to calculate hepatic CL_{int} values according to the following equation and with the use of species-specific scale-up factors for liver weight (grams) per total body weight (kg):

$$CL_{int} = \frac{0.693}{in\ vitro\ t_{1/2}}\ x\ \frac{1\ mL\ incubation}{0.5\ mg\ microsomes}\ x\ \frac{45\ mg\ microsomes}{1\ gram\ liver}\ x\ \frac{x^a\ gram\ liver}{kg\ body\ wt}$$

[a]Scale-up factors used are 45 (rat) and 20 (human).[1]

Predicted hepatic clearance (CL_{hep}) was calculated using the following equation:

$$CL_{hep} = \frac{Q_h * CL_{int}}{Q_h + CL_{int}}$$

Q_h represents hepatic blood flow (mL/min/kg): 21 for human, 70 for rat, and 90 for mouse.

<u>LC-MS/MS Analysis</u>

Prepared samples were injected (10 μL each) onto an AB Sciex Triple Quad 4500 mass spectrometer system with an Agilent 1260 Infinity II pump and autosampler. Mass spectrometer conditions are described in **Table S1.** Quantitation of compounds was performed via AB Sciex Multiquant software using the raw analyte:IS peak area ratios. The typical detection range was 0.5 ng/mL to ≥ 5,000 ng/mL utilizing a quadratic equation regression with 1/x2 weighting.

Correction for dilution of all brain samples (in extraction buffer and subsequently in blank plasma, as previously described) was performed post-quantitation. The corrections for dilution in extraction buffer employed correction factors specific to each brain weight (not shown).

Table S1. LC-MS/MS Conditions*

Injection volume	10 µL	
Mobile phase A	0.5% Formic Acid in Water	
Mobile phase B	0.5% Formic Acid in Acetonitrile	
Flowrate	0.5 mL/min	
Gradient	Time	% Mobile Phase B
	0.0	5
	0.2	5
	0.8	95
	1.5	95
	1.7	5
	2.7	Stop
Column	Fortis C18 (50 x 3.0 mm, 3 µm)	
Data collection and analysis software/version	Analyst v. 1.7.1	
Ionization mode	Positive Electrospray	
Curtain gas (psi)	40	
GS1 (psi)	40	
GS2 (psi)	40	
Capillary voltage (V)	5500	
Source TurboIonSpray® temp. (°C)	500	

HR-MS Spectra of Key Compounds:

VU6035386

**Data File**	VU6035386_0001.d	**Sample Name**	VU6035386
**Sample Type**	Sample	**Position**	P1-C1
**Instrument Name**	Q-TOF	**User Name**	Christopher Presley
**Acq Method**	Auto MSMS_1x50_5-95_90sec.m	**Acquired Time**	1/9/2025 11:48:23 AM (UTC-06:00)
**IRM Calibration Status**	Success	**DA Method**	Default Report AMM_210224.m
**Comment**	3.0 µL Injection		

**Sample Group**		**Info.**	
**Molecular Formula**	C19H13FN4OS	**Stream Name**	LC 1
**Acquisition Time (Local)**	1/9/2025 11:48:23 AM (UTC-06:00)	**Acquisition SW Version**	6200 series TOF/6500 series Q-TOF 10.1 (48.0)
**QTOF Driver Version**	10.01.00	**QTOF Firmware Version**	10.811
**DDE Mode**	2	**Tune Mass Range Max.**	3200

Compound Table

Compound Label	RT	$Mass_{m/z - addt}$	Abund	Name	Formula	$Mass_{TGT}$	Diff (ppm)	Hits (DB)
Cpd 1: VU6035386; C19 H13 F N4 O S;	1.285	364.0797	241474	VU6035386	C19 H13 F N4 O S	364.0794	0.8	1

Compound Label	Name	$m/z_{Observed}$	RT	Algorithm
Cpd 1: VU6035386; C19 H13 F N4 O S;	VU6035386	365.0870	1.285	Find by Formula

Calc m/z	Ion
365.0867	(M+H)+

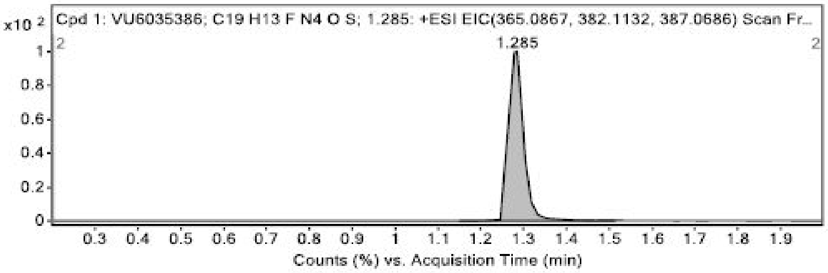

MS Zoomed Spectrum

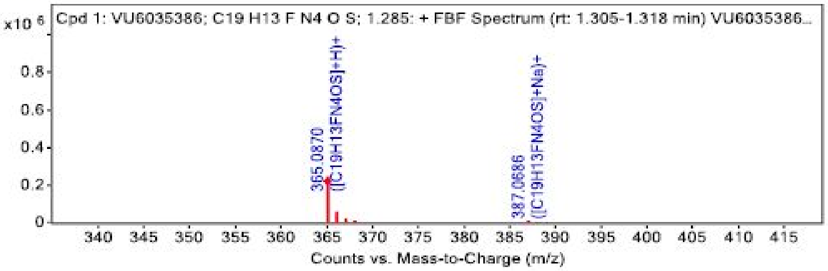

MS Spectrum Peak List

m/z	z	Abund	Formula	Ion
365.0870	1	241474.36	C19H13FN4OS	(M+H)+
366.0897	1	49428.43	C19H13FN4OS	(M+H)+
367.0877	1	12396.19	C19H13FN4OS	(M+H)+
368.0885	1	2289.78	C19H13FN4OS	(M+H)+
387.0686	1	5390.3	C19H13FN4OS	(M+Na)+
388.0697	1	1481.13	C19H13FN4OS	(M+Na)+
389.0651	1	435.09	C19H13FN4OS	(M+Na)+

MS Zoomed Spectrum

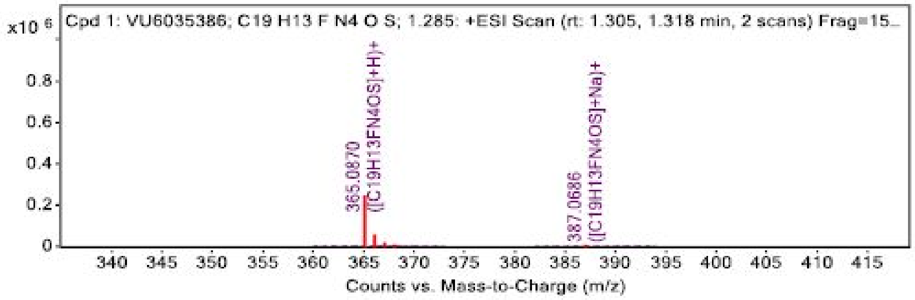

MS Spectrum Peak List

m/z	Calc m/z	Diff(ppm)	z	Abund	Formula	Ion
365.0870	365.0867	0.76	1	241474.36	C19H13FN4OS	(M+H)+
366.0897	366.0895	0.37	1	49428.43	C19H13FN4OS	(M+H)+
367.0877	367.0862	4.05	1	12396.19	C19H13FN4OS	(M+H)+
368.0885	368.0872	3.56	1	2289.78	C19H13FN4OS	(M+H)+
387.0686	387.0686	0.03	1	5390.3	C19H13FN4OS	(M+Na)+
388.0697	388.0715	-4.44	1	1481.13	C19H13FN4OS	(M+Na)+
389.0651	389.0681	-7.79	1	435.09	C19H13FN4OS	(M+Na)+

MSMS Spectrum

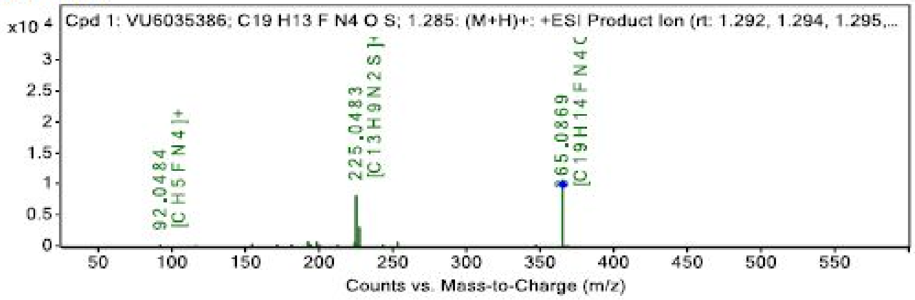

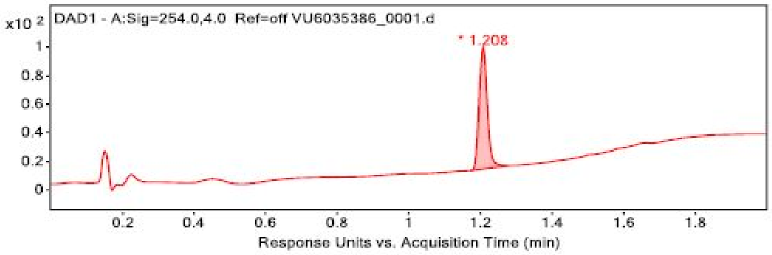

User Chromatogram Peak List

RT	Height	Normalized Height	Height %	Area	Area %	Area Sum %	Symmetry	Width
1.208	30	84.98	100	47.69	100	100	1.23	0.103

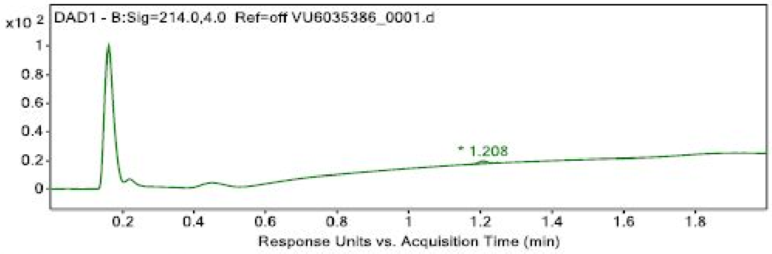

User Chromatogram Peak List

RT	Height	Normalized Height	Height %	Area	Area %	Area Sum %	Symmetry	Width
1.208	18.81	2.03	100	29.38	100	100	0.74	0.073

--- End Of Report ---

VU6035474

**Data File**	VU6035474_0001.d	**Sample Name**	VU6035474
**Sample Type**	Sample	**Position**	P1-C2
**Instrument Name**	Q-TOF	**User Name**	Christopher Presley
**Acq Method**	Auto MSMS_1x50_5-95_90sec.m	**Acquired Time**	1/9/2025 11:55:02 AM (UTC-06:00)
**IRM Calibration Status**	Success	**DA Method**	Default Report AMM_210224.m
**Comment**	3.0 µL Injection		

**Sample Group**		**Info.**	
**Molecular Formula**	C20H16FN5O	**Stream Name**	LC 1
**Acquisition Time (Local)**	1/9/2025 11:55:02 AM (UTC-06:00)	**Acquisition SW Version**	6200 series TOF/6500 series Q-TOF 10.1 (48.0)
**QTOF Driver Version**	10.01.00	**QTOF Firmware Version**	10.811
**DDE Mode**	2	**Tune Mass Range Max.**	3200

Compound Table

Compound Label	RT	$Mass_{m/z - addt}$	Abund	Name	Formula	$Mass_{TGT}$	Diff (ppm)	Hits (DB)
Cpd 1: VU6035474; C20 H16 F N5 O; 1.155	1.155	361.1337	426185	VU6035474	C20 H16 F N5 O	361.1339	-0.57	1

Compound Label	Name	$m/z_{Observed}$	RT	Algorithm
Cpd 1: VU6035474; C20 H16 F N5 O; 1.155	VU6035474	362.1409	1.155	Find by Formula

Calc m/z	Ion
362.1412	(M+H)+

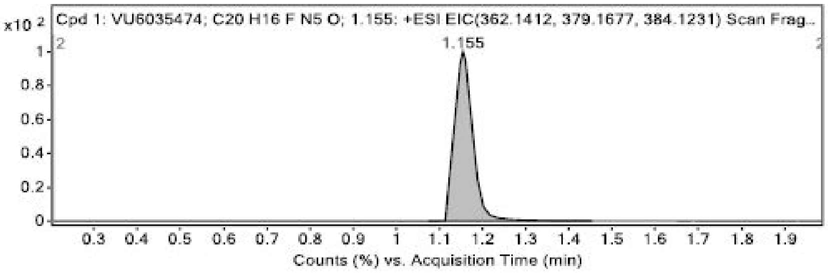

MS Zoomed Spectrum

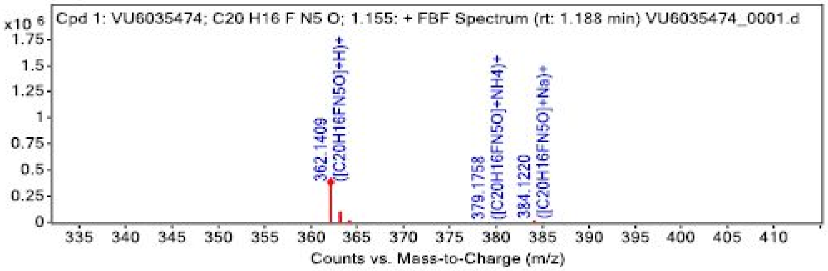

MS Spectrum Peak List

m/z	z	Abund	Formula	Ion
362.1409	1	426185.19	C20H16FN5O	(M+H)+
363.1444	1	91776.7	C20H16FN5O	(M+H)+
364.147	1	12232.74	C20H16FN5O	(M+H)+
379.1758	1	320.56	C20H16FN5O	(M+NH4)+
384.122	1	12416.57	C20H16FN5O	(M+Na)+
385.1244	1	2719.34	C20H16FN5O	(M+Na)+

MS Zoomed Spectrum

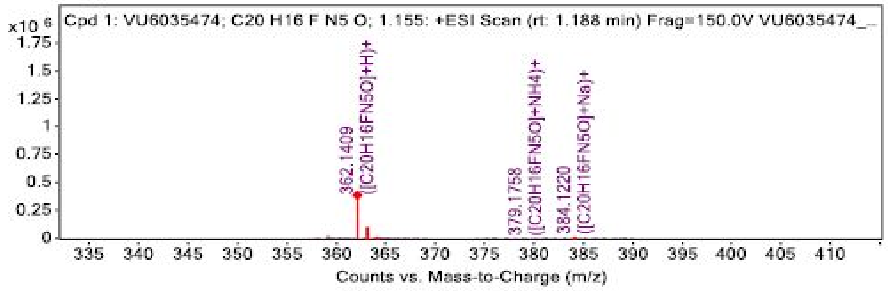

MS Spectrum Peak List

m/z	*Calc m/z*	Diff(ppm)	z	Abund	Formula	Ion
362.1409	362.1412	-0.83	1	426185.19	C20H16FN5O	(M+H)+
363.1444	363.1441	0.85	1	91776.7	C20H16FN5O	(M+H)+
364.1470	364.1468	0.63	1	12232.74	C20H16FN5O	(M+H)+
379.1758	379.1677	21.43	1	320.56	C20H16FN5O	(M+NH4)+
384.1220	384.1231	-2.77	1	12416.57	C20H16FN5O	(M+Na)+
385.1244	385.1260	-4.24	1	2719.34	C20H16FN5O	(M+Na)+

MSMS Spectrum

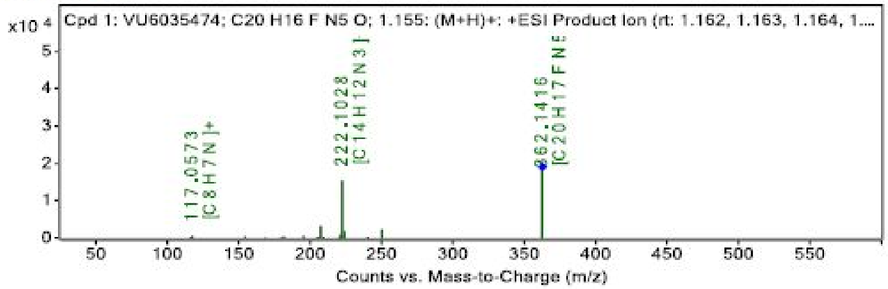

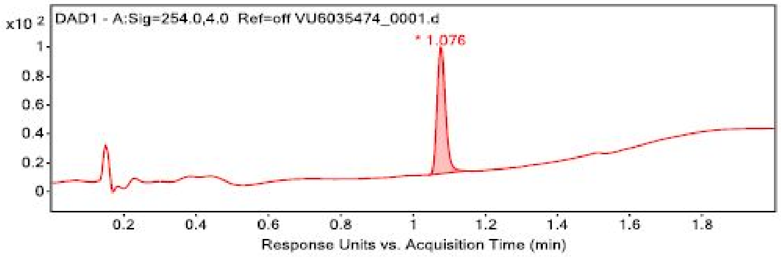

User Chromatogram Peak List

RT	Height	Normalized Height	Height %	Area	Area %	Area Sum %	Symmetry	Width
1.076	26.53	87.32	100	42.83	100	100	1.53	0.103

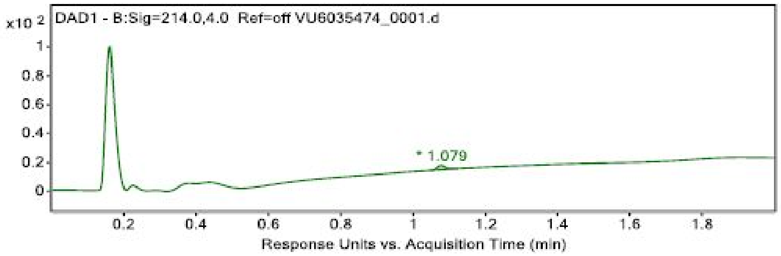

User Chromatogram Peak List

RT	Height	Normalized Height	Height %	Area	Area %	Area Sum %	Symmetry	Width
1.079	28.41	2.83	100	46.21	100	100	0.98	0.077

--- End Of Report ---

^{1}H and ^{13}C NMR Spectra of Key Compounds:

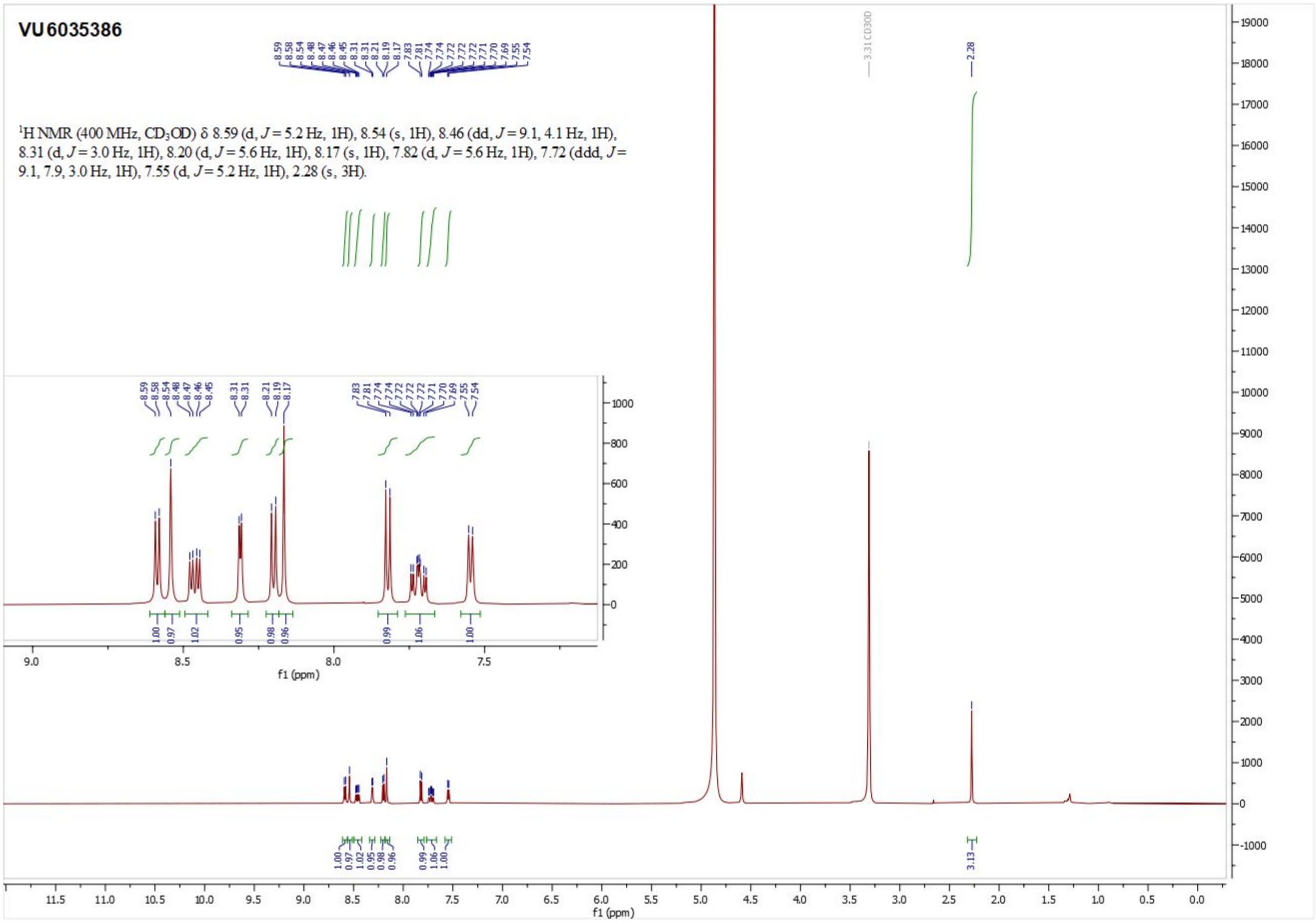

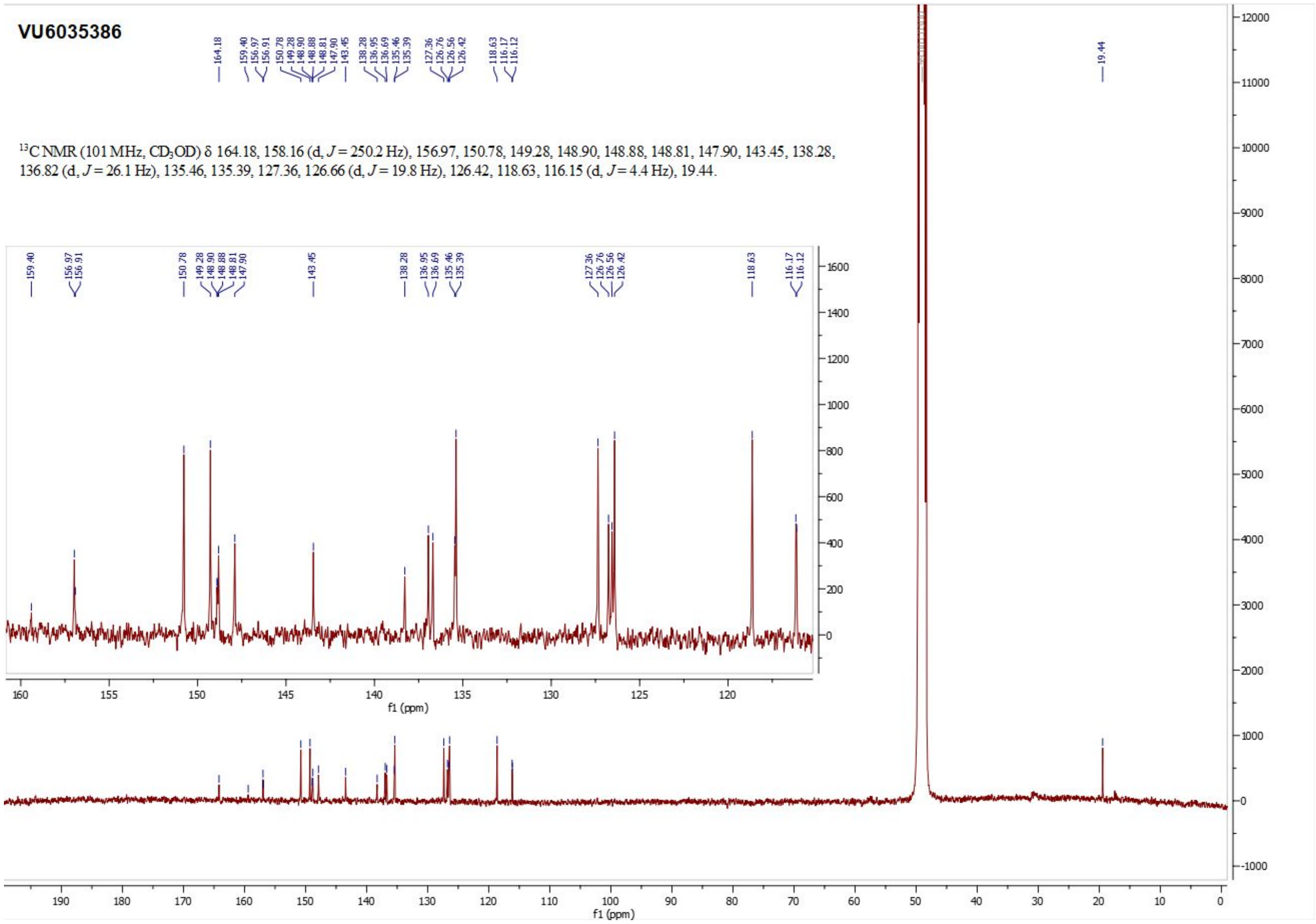

VU6035386
^{13}C NMR (101 MHz, CD_3OD) δ 164.18, 158.16 (d, J = 250.2 Hz), 156.97, 150.78, 149.28, 148.90, 148.88, 148.81, 147.90, 143.45, 138.28, 136.82 (d, J = 26.1 Hz), 135.46, 135.39, 127.36, 126.66 (d, J = 19.8 Hz), 126.42, 118.63, 116.15 (d, J = 4.4 Hz), 19.44.
f1 (ppm)
f1 (ppm)

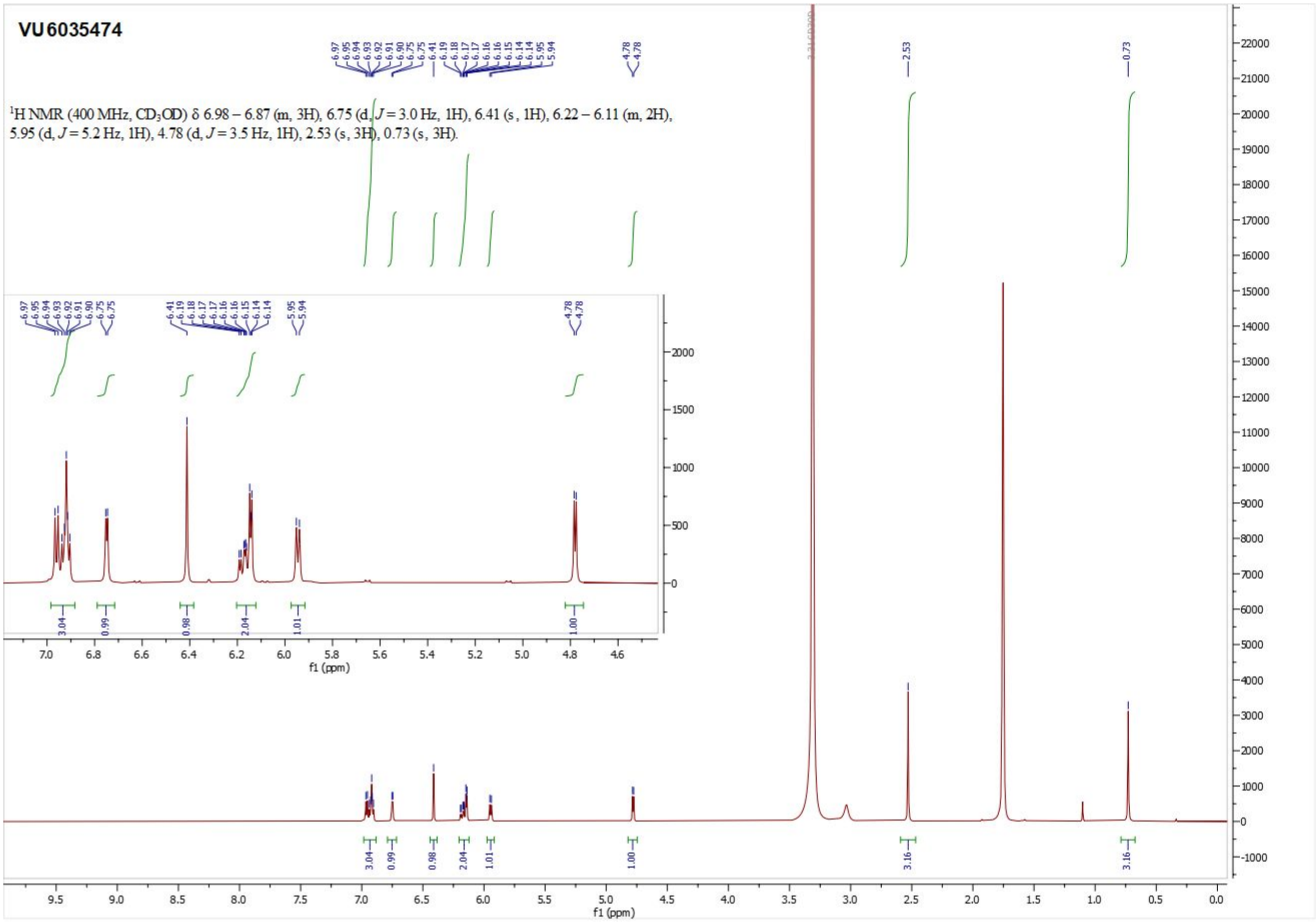
VU6035474
1H NMR (400 MHz, CD3OD) δ 6.98 – 6.87 (m, 3H), 6.75 (d, J = 3.0 Hz, 1H), 6.41 (s, 1H), 6.22 – 6.11 (m, 2H),
5.95 (d, J = 5.2 Hz, 1H), 4.78 (d, J = 3.5 Hz, 1H), 2.53 (s, 3H), 0.73 (s, 3H).
f1 (ppm)

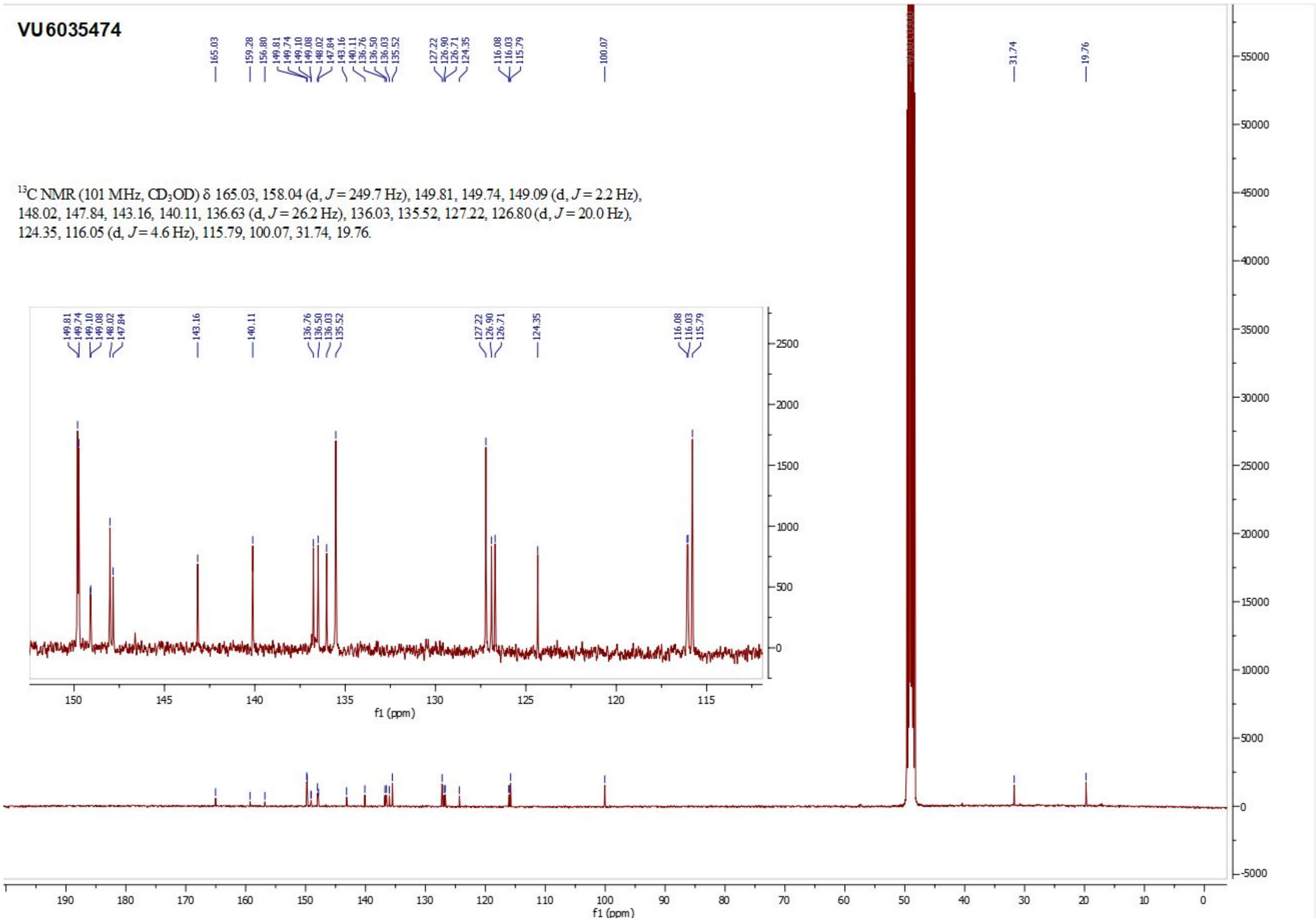

References

1) Lin J.H.; Chiba M.; Balani S.K; Chen I.W.; Kewi G.Y.; Vastag K.J.; Nishime J.A. Species differences in the pharmacokinetics and metabolism of indinavir, a potent human immunodeficiency virus protease inhibitor. *Drug Metab Dispos*. 1996, 24, 1111-1120.
